# Supplementary material for: In-situ-sprayed therapeutic hydrogel for oxygen-actuated Janus regulation of postsurgical tumor recurrence/metastasis and wound healing
Source: Nat Commun. 2024 Jan 27;15:814. doi: 10.1038/s41467-024-45072-x (PMC10821930; doi:10.1038/s41467-024-45072-x)
Supplement: Supplementary file 1 — Supplementary Information file [file 41467_2024_45072_MOESM1_ESM.pdf]

Supplementary Information

In-situ-sprayed therapeutic hydrogel for oxygen-actuated Janus regulation of postsurgical tumor recurrence/metastasis and wound healing

Shuiling Chen[1,2], Yang Luo[1,2], Yang He[1,2], Ming Li[1,2], Yongjian Liu[1,2], Xishen Zhou[1,2], Jianwen Hou[1,2*] & Shaobing Zhou[1,2*]

[1]Institute of Biomedical Engineering, College of Medicine, Southwest Jiaotong University, Chengdu, 610031, China

[2]Key Laboratory of Advanced Technologies of Materials Ministry of Education, School of Materials Science and Engineering, Southwest Jiaotong University, Chengdu, 610031, China

*Corresponding authors.

E-mail: houjianwen@swjtu.edu.cn; shaobingzhou@swjtu.edu.cn

1. Methods

Chemicals and reagents

Zinc nitrate hexahydrate ($Zn(NO_3)_2$ $6H_2O$) and Dihydrorhodamine 123 (DHR) were obtained from Aladdin Industrial Corporation. 2-methylimidazole (2-MIM), L-Arginine (L-Arg), indocyanine green (ICG) and 1,3-diphenylisobenzofuran (DPBF) were acquired from Adamas-beta. Sodium alginate and calcium chloride anhydrate ($CaCl_2$) were obtained from Chengdu Kelong Chemical Co., Ltd. 2,7-dichlorodihydrofluorescein diacetate (DCFH-DA) and NO fluorescent probe (DAF-FM DA) were obtained from Beyotime Biotechnology. GSH detection reagent (ThiolTracker Violet) was obtained from Invitrogen Thermo Fisher Scientific. ROS frozen section staining kit, NO frozen section staining kit and peroxynitrite frozen section staining kit were obtained from BestBio.

Material characterizations

The sample morphologies were characterized by transmission electron microscopy (TEM) (JEM-2100F, Japan) and scanning electron microscopy (SEM) (JSM 7800F, Japan). The hydrodynamic size and surface zeta potential were tested by dynamic light scattering (DLS) (ZETA-SIZER, MALVERN NanoZS90, Malvern, Ltd., UK). The ultraviolet-visible (UV-vis) absorption spectra were measured by UV-2550 spectrometer (Shimadzu, Japan). The chemical composition was characterized with fourier transform infrared spectroscopy (FT-IR, Fluke, Ti480). The crystalline structure of nanoparticles was analyzed using Empyrean X-ray diffractometer (PANalytical B.V., NED). Fluorescence spectrum was recorded by an RF-7000 fluorescence spectrophotometer (Shimadzu, Kyoto, Japan). The concentrations of dissolved O_2 were measured by a dissolved O_2 meter (Rex, JPBJ-608).

Thermal gravimetric analysis (TGA)

TGA was performed on a thermal analyzer (Mettler Toledo TGA/DSC 3+, Switzerland) as follows. About 10 mg of different samples (ZIF-8, L@Z, IL@Z and HIL@Z) were separately filled into alumina crucible and heated under a nitrogen flow increasing from 30 ℃ to 800 ℃ at a ramp rate of 10 ℃/min. TGA was then conducted by the weight change associated with

the temperature program in a controlled atmosphere.

In vitro ICG release

The release of ICG from nanoparticles or hydrogels in phosphate buffered saline (PBS) with different pH values (5.5 and 7.4) was monitored under stirring (100 rpm). The cumulative quantity of ICG released from nanoparticles or hydrogels was determined based on the UV-vis absorption intensity at 780 nm.

Determination of ROS, NO and $ONOO^-$ generation

DPBF was used as the $^1O_2$-trapping agent to characterize ROS produced by HIL@Z nanoparticles under 808 nm irradiation (0.0, 1.0 and 1.5 W/cm^2) after different time. Briefly, DPBF was dissolved in methanol in the dark to obtain the solution with a concentration of 20 μM. Then HIL@Z nanoparticles dispersed in PBS (1 mg/mL) were transferred into DPBF solution. After 808 nm irradiation with a certain time, the supernatants were collected and the absorption at 410 nm of vestigial DPBF was determined by an UV-vis spectrophotometer.

The NO release from HIL@Z nanoparticles (1.0 mg/mL) was checked using Griess reagent kit under 808 nm irradiation (0.0 and 1.5 W/cm^2) for 10 min. To quantify the generated NO concentration, commercial $NaNO_2$ (0.8-100 μM) was first used to establish a standard curve. HIL@Z nanoparticles were uniformly dispersed in PBS (2.0 mL). After 808 nm irradiation with a certain time, the supernatant (50 μL), Griess Reagent I (50 μL) and Griess Reagent II (50 μL) were successively added into the 96-well plates. Then the absorption at 540 nm was measured by the automated microplate spectrophotometer.

DHR, which could produce strong green fluorescence at 524 nm (Ex: 505 nm), was employed as a fluorescent probe for studying the $ONOO^-$-generating ability of HIL@Z nanoparticles. The fluorescence spectra of HIL@Z or HI@Z mixed with DHR were measured before and after 808 nm irradiation (1.5 W/cm^2, 0-10 min). Vitamin C (60 μM), the free radical scavenger, was evenly dispersed into the mixed solution to assess its quenching performance toward superoxide anion free radical.

Cell viability assay

The cytocompatibility of the materials was evaluated through live/dead staining and alamar blue (AB) assay. For the live/dead staining, B16F10 cells and HUVECs (1×10^5 cells/well) were incubated in 12-well plates at 37 ℃ for 24 h. Then the cells were incubated with different concentrations of materials for additional 24 h. After washing with PBS for three times, the mixed solution of calcein acetoxymethylester (Calcein-AM) and propidium iodide (PI) was added into all the wells and the cells were stained at 37 ℃ for 15 min. Live and dead cells were stained green and red, respectively. For AB detection, B16F10 cells and HUVECs (2×10^4 cells/well) were incubated in 48-well plates at 37 ℃ for 24 h. After that, the cells were incubated with different concentrations of materials for additional 24 h. All the wells were washed by PBS for three times. Then 300 μL of AB detection solution (80% medium 199 (M199): 10% fetal bovine serum (FBS): 10% AB, v/v) was added into each well. After incubation for 3 h, 200 μL of AB solution in each well was added into the 96-well plates, and the absorbance at 570 nm was determined by a microplate reader.

Cellular uptake behavior

B16F10 cells (5×10^5 cells/well) were inoculated in a 6-well plate and cultured for 24 h. Afterwards, cells were cultured in the medium containing different nanoparticles (BL@Z, HBL@Z and HBL@Z+HA). After incubating for further 2.5 h, cells were washed with fresh PBS thoroughly and observed by fluorescence microscopy. Furthermore, the cellular uptake of different nanoparticles by B16F10 cells was also studied by flow cytometry.

Lysosomal escape behavior

B16F10 cells (5×10^5 cells/well) were inoculated in a 6-well plate and cultured for 24 h. The adhered B16F10 cells were incubated with HBL@Z for 2.5 h and 4 h, respectively. Then B16F10 cells were washed three times with PBS before staining, and lysosomes were labeled with the Lyso-Tracker Green (Beyotime Biotechnology, C1047S). The fluorescence signal was observed by fluorescence microscopy.

Western blot (WB) analysis

B16F10 cells were inoculated in 6-well plates at a density of 5×10^5 cells per well containing 2 mL of RPMI 1640 and cultured for 24 h. Then the culture medium was removed and cells were treated under different conditions in the dark for 2.5 h. The cells were irradiated with 635 nm or 808 nm laser for 20 min. After continuous culture for 24 h, the cells were collected and lysed in RIPA lysis buffer (Beyotime, P0013) for total protein extraction. Subsequently, protein concentrations were measured by pierceTM rapid gold BCA protein assay kit (Thermo Fisher, A53225), separated on 10% SDS PAGE, and transferred onto PVDF membrane (MILLIPORE, IPVH00010). Next, PVDF membranes were blocked by TBST containing 5% skim milk for 1 h. After that, the PVDF membranes were washed with TBST and incubated with various primary antibodies (listed in Supplementary Table 1) overnight at 4°C. Then, the PVDF membranes were washed with TBST and incubated with secondary antibodies HRP-goat anti rabbit (Biosharp, BL003A) at room temperature for 1 h. After washing with TBST for five times, protein detection was visualized using an enhanced chemiluminescence (ECL) kit (Monna, QuickChemi 5100).

Real-time quantitative polymerase chain reaction (RT-qPCR) analysis

For RT-qPCR analysis, B16F10 cells, HUVECs and L929 cells were respectively inoculated in 6-well plates at a density of 5×10^5 cells per well containing 2 mL of fresh medium. After culturing for 24 h, the culture medium was removed and cells were treated under different conditions in the dark for 30 min. The cells were irradiated with 635 nm or 808 nm laser for 20 min. After continuous culture for 24 h, the cells were collected and harvested for RNA extraction through a total RNA extraction kit (Janstone Bio, TR205-50). Subsequently, first strand cDNA was synthesized through a 5X All-in-one MasterMix (with AccuRT Genomic DNA removal kit) (Abm, #G492) according to the manufacturer's instructions. After that, the prepared cDNAs were mixed with specific primers (listed in Supplementary Table 2) and EvaGreen express 2X qPCR MasterMix-No Dye (Abm, 0194844830001). And the expression levels of related genes including *HIF-1α, IL-6* and *β-actin* were detected by RT-qPCR (BIOER, FQD-96C). The amplification was performed by a fluorescence detection system

with Evagreen fluorescence for 40 cycles. Each cycle consisted of thermal denaturation at 95 ℃ for 15 s and annealed at 60 ℃ for 60 s. Finally, each sample was quantified by comparing the Livak ($2^{-\Delta\Delta CT}$) method and relative gene expression was calculated using the reference gene *β-actin*.

In vitro cytotoxicity assay

The cytotoxicity against B16F10 cells was assessed by AB assay. B16F10 cells (2×10^4 cells/well) were inoculated in a 48-well plate and cultured for 24 h. Then the cells were subjected to different treatments (Control, Red+NIR, HIL@Z/P/H, HL@Z/P/H+NIR, HI@Z/P/H+NIR, HIL@Z/P/H+Red, HIL@Z/P/H+NIR, HIL@Z/P/H+Red+NIR) for 4 h. During the incubation period, the cells were irradiated with 635 nm or 808 nm laser for 20 min. After continuous culture for 12 h, the survival rate of cells was calculated by the method similar to the cell viability assay.

B16F10 cells (5×10^5 cells/well) were inoculated in 6-well plates. After 24 h incubation, the cells were subjected to different treatments (Control, Red+NIR, HIL@Z/P/H, HL@Z/P/H+NIR, HI@Z/P/H+NIR, HIL@Z/P/H+Red, HIL@Z/P/H+NIR, HIL@Z/P/H+Red+NIR) for 4 h. During the incubation period, the cells were irradiated with 635 nm or 808 nm laser for 20 min. After staining by Calcein-AM (10 μM) and PI (1 μM) for 10 min, the cell-killing effect was verified using fluorescence microscopy.

B16F10 cells were seeded in 6-well plates. After 24 h incubation, the cells were subjected to different treatments (Control, Red+NIR, HIL@Z/P/H, HL@Z/P/H+NIR, HI@Z/P/H+NIR, HIL@Z/P/H+Red, HIL@Z/P/H+NIR, HIL@Z/P/H+Red+NIR) for 4 h. During the incubation period, the cells were irradiated with 635 nm or 808 nm laser for 20 min. With reference to the instructions of the AnnexinV-APC/7-AAD cell apoptosis detection kit (Multi Sciences, AT105-100), the percentage of apoptotic cells was assessed by flow cytofluorometry analysis after staining with AnnexinV-APC (5 μL) and 7-AAD (10 μL) dyes for 5 min.

Hemolysis test and whole blood clotting test

To determine the blood compatibility of HIL@Z/P/H, the hemolysis and whole blood clotting study were carried out. Whole blood was collected from healthy C57BL/6 mice. The purified

red blood cells (RBCs) were obtained by centrifuging and diluted to a concentration of 2% (v/v). Next, RBCs suspended in PBS solution (0.5 mL) were mixed with PBS solution (0.5 mL) containing 10^4, 10^5, 10^6, 10^7, 10^8 and 10^9 cells/mL of HIL@Z/P/H. Deionized (DI) water and PBS buffer were set as the positive and negative controls, respectively. The supernatant was collected with centrifugation after 1 h incubation at 37 ℃. Afterward, the absorbance of the supernatant was measured with a microplate reader (Varioskan LUX, Thermo Scientific, USA) at 540 nm. The hemolysis rate was calculated using the following equation:

$$\text{Hemolysis (\%)} = \frac{A_s - A_n}{A_p - A_n} \times 100\% \qquad (1)$$

Where A_s, A_n and A_p are the absorbance values of the supernatant fraction of the samples, negative (PBS) and positive (DI water) controls, respectively.

The whole blood solution (50 μL) was added into polypropylene tubes containing HIL@Z/P/H (0.5 mL) with different concentrations (10^4, 10^5, 10^6, 10^7, 10^8 and 10^9 cells/mL) to initiate coagulation. Whole blood (50 μL) in DI water and PBS was used as the positive and negative controls, respectively. After 90 s, 10 mL of DI water was gently added into polypropylene tubes without disturbing the clot. The supernatant was collected with centrifugation. Afterward, the absorbance of the supernatant was measured with a microplate reader (Varioskan LUX, Thermo Scientific, USA) at 540 nm. The blood clotting index was calculated according to following equation:

$$\text{Blood clotting indox (\%)} = \frac{A_s}{A_p} \times 100\% \qquad (2)$$

Where A_s represents the absorbance of sample and A_p represents the absorbance of the reference value (positive control).

In vitro HUVEC proliferation assay

HUVECs (2×10^4 cells/well) were seeded into lower chambers of 48-well transwell inserts with 0.4 μm pore-sized filters (Corning, USA) and cultured for 24 h. Then the control (PBS) was cultured without upper chamber, while other groups were cultured with upper chambers containing hydrogel, P/H, P/H+GW4869 and P/H+Red, respectively. After incubation for 24 h, all the wells were washed by PBS for three times. Then 300 μL of AB detection solution was added into per well. After incubation for 3 h, 200 μL of AB solution in each well was

transfered to the 96-well plates. And the OD_{680} values was determined using a microplate reader to assess the cell viability.

In vitro HUVEC migration assay

HUVECs (6×10^4 cells/well) were seeded into lower chambers of 12-well transwell inserts with 0.4 μm pore-sized filters. When the cell confluence reached about 80%, they were scratched with a plastic tip (200 μL). Then the cells were washed with PBS to remove the unattached cells. Then fresh serum-free DMEM/F-12 medium was added and the attached cells were treated with control (PBS), hydrogel, P/H, P/H+GW4869, and P/H+Red in the upper chamber, respectively. HUVECs were photographed at 0, 6, 12, and 24 h after wounding, the healing area was calculated by Image J (version 2.1.0) software and calculated according to the following formula:

$$\text{Relative migration ratio (\%)} = \frac{M_0\text{-}M_t}{M_0} \times 100\% \qquad (3)$$

Where M_0 represents the initial scratch area and M_t represents the final scratch area.

In vitro HUVEC tube formation assay

For tube formation assay, 150 μL of the diluted Matrigel was vertically added in the center of the pre-cooled 24-well transwell plate lower chamber and incubated at 37 ℃ for 30 min to form a gel. Subsequently, HUVECs (5×10^4 cells/well) were seeded into lower chambers. The upper chambers were coated with control (PBS), hydrogel, P/H, P/H+GW4869 and P/H+Red, respectively. After 8 h of incubation, tube formation was observed using fluorescence microscopy and quantitated by Image J software.

Statistical analysis

All measurements were performed on three or more independent replicates from separate experiments. The exact sample size and statistical test for each experiment were described in the relevant figure legends. All results were presented as the mean ± SD. All statistical data were processed in Origin 2021 (version 9.8.0.200) by Student’s t-test and one-way ANOVA.

2. Supplementary Tables

Supplementary Table 1 Antibodies used for this work.

Antibodies	Provider	Catalog	Dilution used
Anti-HIF-1 alpha antibody (HIF-1α)	Abcam	#ab16066	1/2000 dilution
Anti-MMP-9 antibody (MMP-9)	Servicebio	#GB11132-100	1/1000 dilution
Anti-EPO antibody (EPO)	Abcam	#ab271007	1/1000 dilution
Anti-Heme Oxygenase 1 antibody (HO-1)	Abcam	#ab189491	1/2000 dilution
Adrenomedullin Polyclonal antibody (ADM)	Proteintech	#10778-1-AP	1/1000 dilution
Anti-GLUT1 antibody (Glut1)	Abcam	#ab115730	1/5000 dilution
Anti-beta Actin antibody (β-actin)	ZENBIO	380624	1/1000 dilution
Anti-VEGF Receptor 2 antibody (VEGF)	Abcam	#ab115805	1/100 dilution
Goat Anti-Rabbit IgG	Biosharp	#BL003A	1/40000 dilution
Anti-CD31 antibody (CD31)	Abcam	#ab182981	1/1000 dilution
Anti-alpha smooth muscle Actin antibody (α-SMA)	Abcam	#ab7817	1/100 dilution
Anti -CD3 antibody (CD3)	Servicebio	#GB11014-100	1/500 dilution
Anti-CD8 alpha antibody (CD8)	Abcam	#ab217344	1/2000 dilution
Anti-F4/80 antibody (F4/80)	Proteintech	#28463-1-AP	1/100 dilution

Supplementary Table 2 Primers used for this work..

Gene	Species	NCBI gene ID	NCBI search number	Oligo name	Sequence 5′-3′
HIF-1α	Mouse	15251	NM_010431.3	*HIF-1α*-F	AGTGCTGATCCTGCACTGAA
				HIF-1α-R	AGGCTGGGAAAAGTTAGGAGTG
IL-6	Human	3569	XM_054358146.1	*IL-6*-F	TTCGGTCCAGTTGCCTTCTC
				IL-6-R	TGTTTTCTGCCAGTGCCTCT
IL-6	Mouse	16193	NM_031168.2	*IL-6*-F	AGTTCCTCTCTGCAAGAGACTTCC
				IL-6-R	TTGCCATTGCACAACTCTTTTC
β-actin	Human	81822	NM_031144	*β-actin*-F	AATCTGGCACCACACCTTCTACAA
				β-actin-R	GGATAGCACAGCCTGGATAGCAA
β-actin	Mouse	11461	NM_007393	*β-actin*-F	GTGCTATGTTGCTCTAGACTTCG
				β-actin-R	ATGCCACAGGATTCCATACC

3. Supplementary Figures

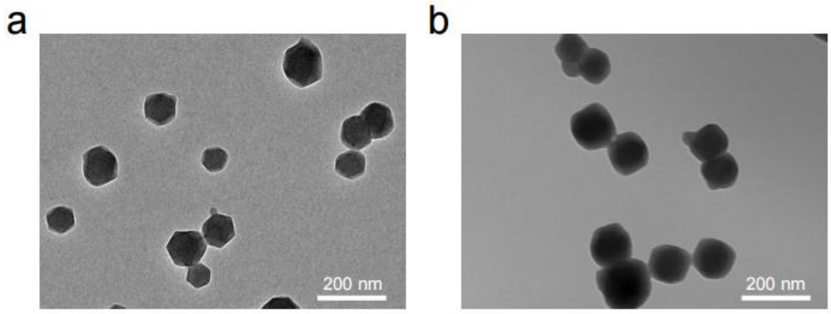

Supplementary Fig. 1 Characterization of nanoparticles. TEM images of (**a**) ZIF-8 and (**b**) IL@Z nanoparticles. The results were representative of three independent experiments.

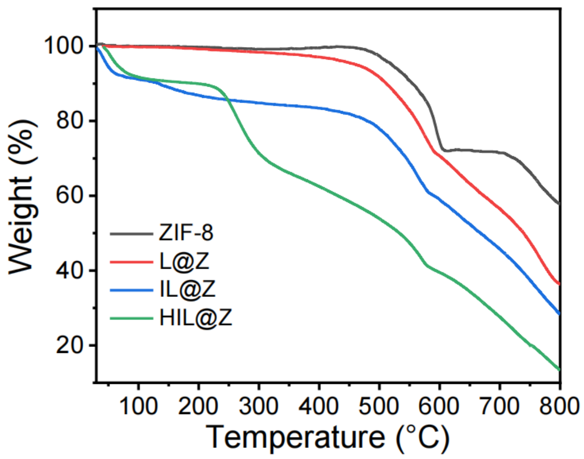

Supplementary Fig. 2 Thermogravimetric curves of different nanoparticles. Source data are provided as a Source Data file.

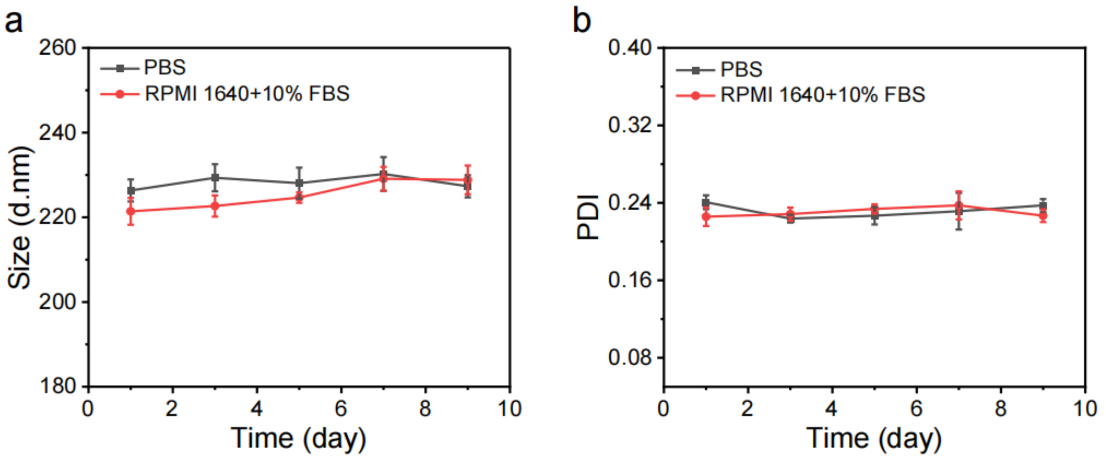

Supplementary Fig. 3 Stability of HIL@Z. The change of (**a**) hydrated particle size and (**b**) polydispersity index (PDI) of HIL@Z after storage in PBS (pH = 7.4) and RPMI 1640 medium with 10% FBS for different days. Data were presented as mean ± SD, n = 3 biologically independent samples. Source data are provided as a Source Data file.

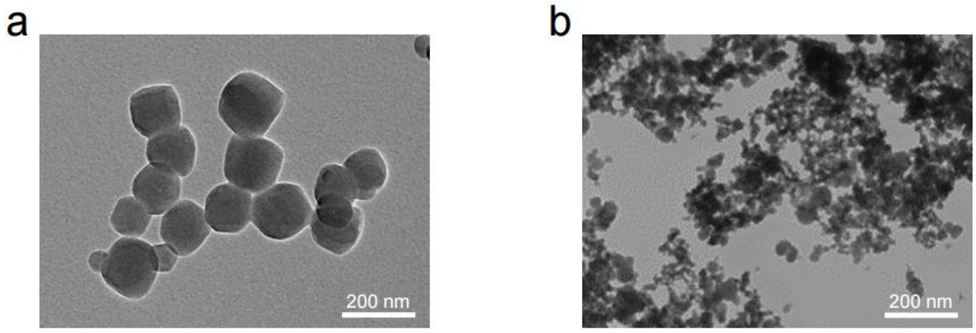

Supplementary Fig. 4 pH-responsive property of HIL@Z. Representative TEM images of HIL@Z immersed in (**a**) PBS buffer (pH = 7.4) and (**b**) PBS buffer (pH = 5.5) for 24 h. The results were representative of three independent experiments.

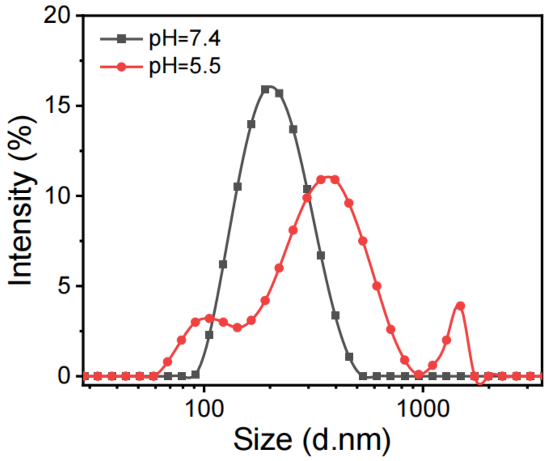

Supplementary Fig. 5 Hydrodynamic particle size distribution of HIL@Z immersed in PBS buffer with pH = 7.4 and pH = 5.5 for 24 h. Source data are provided as a Source Data file.

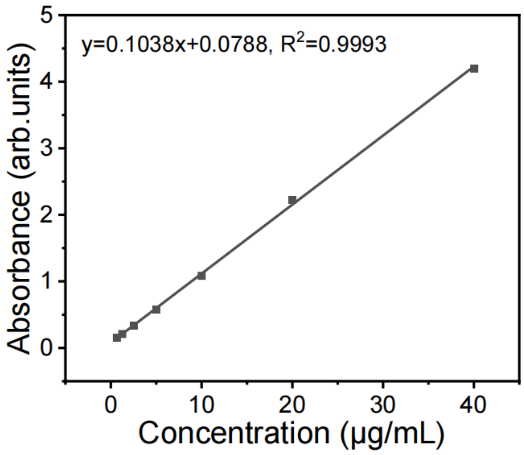

Supplementary Fig. 6 The linear relationship between the UV-vis absorbance intensity at 780 nm and the concentration of ICG. Source data are provided as a Source Data file.

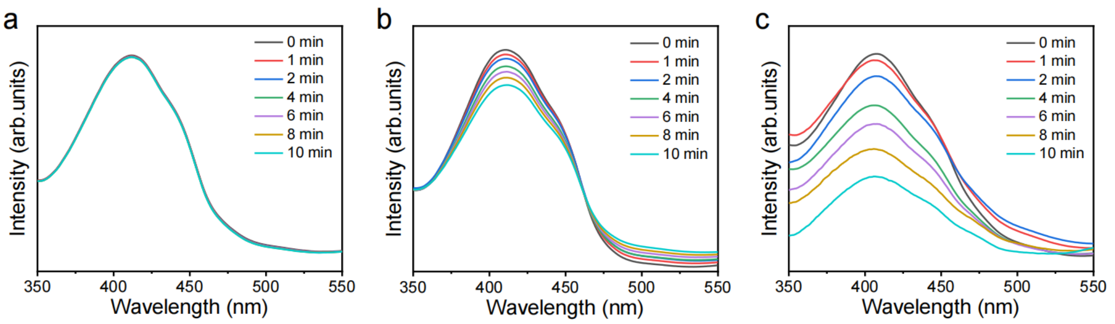

Supplementary Fig. 7 In vitro ROS generation by HIL@Z. Time-dependent absorbance spectra of HIL@Z nanoparticles co-incubated with DPBF under NIR irradiation with various power intensities: (**a**) 0.0 W/cm^2, (**b**) 1.0 W/cm^2, and (**c**) 1.5 W/cm^2. Source data are provided as a Source Data file.

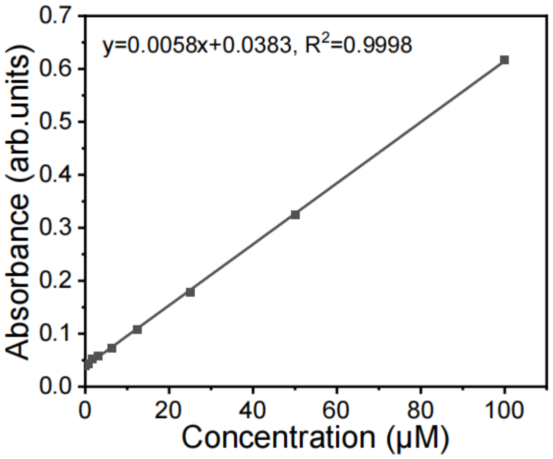

Supplementary Fig. 8 The linear relationship between the UV-vis absorbance intensity at 540 nm and the concentration of NO. Source data are provided as a Source Data file.

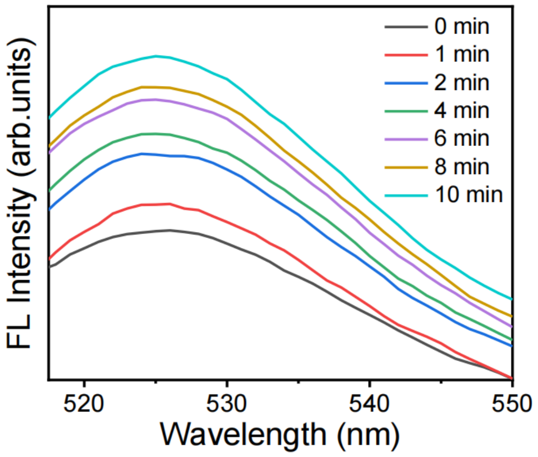

Supplementary Fig. 9 Fluorescence spectrum of $ONOO^-$ detected by DHR after NIR laser irradiation for different time. Source data are provided as a Source Data file.

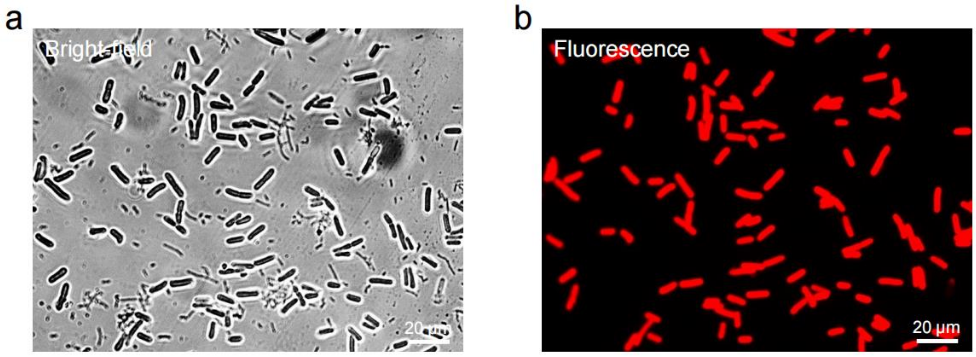

Supplementary Fig. 10 Microscopy morphology of PCC 7942. (**a**) Bright-field and (**b**) fluorescence images of PCC 7942. The results were representative of three independent experiments.

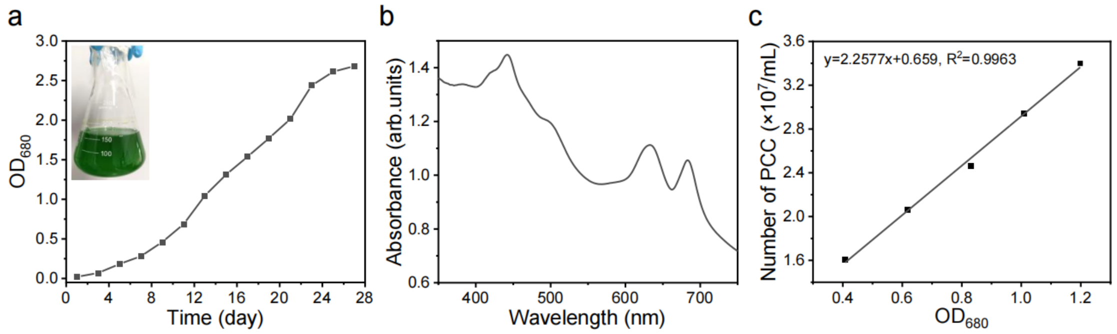

Supplementary Fig. 11 The growth period of PCC 7942. (**a**) The growth curve of PCC 7942 (inset: the image of PCC 7942 culture solution). (**b**) UV-vis absorbance spectra of PCC 7942. (**c**) The corresponding concentration of PCC 7942 to the absorbance at 680 nm. Source data are provided as a Source Data file.

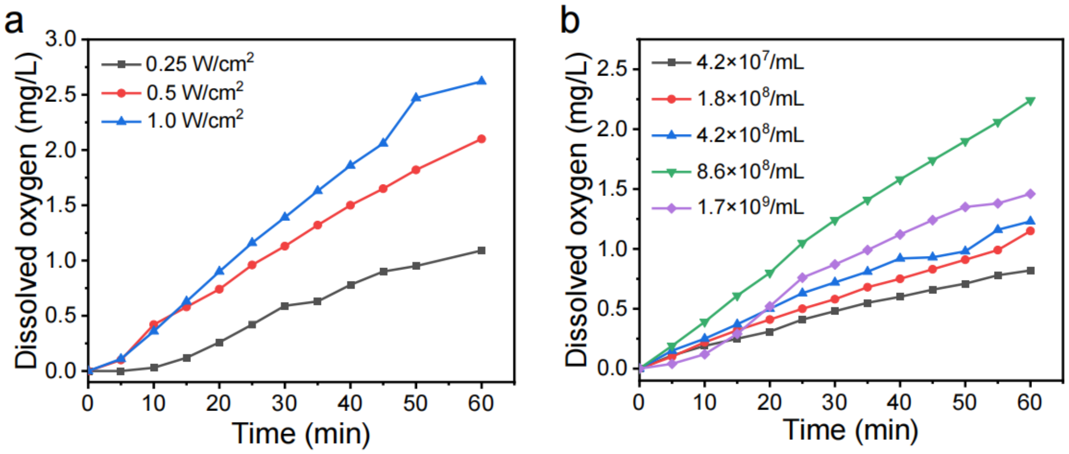

Supplementary Fig. 12 O_2 production performance of PCC 7942. (**a**) Comparison of releasing dissolved O_2 of PCC 7942 under Red irradiation with different power densities. (**b**) Comparison of releasing dissolved O_2 of PCC 7942 with different concentrations. Source data are provided as a Source Data file.

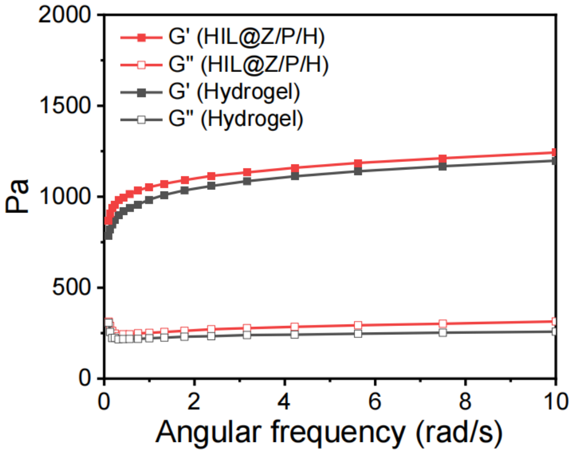

Supplementary Fig. 13 Rheological properties of hydrogel and HIL@Z/P/H. Source data are provided as a Source Data file.

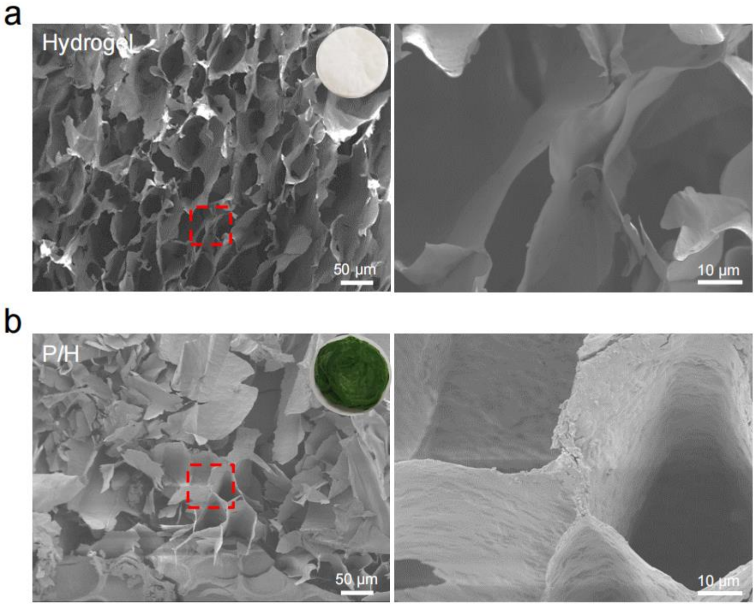

Supplementary Fig. 14 Morphological observation of hydrogel and P/H. SEM images with different magnifications of (**a**) hydrogel and (**b**) P/H (insets: photographs of the lyophilized corresponding samples). The results were representative of three independent experiments.

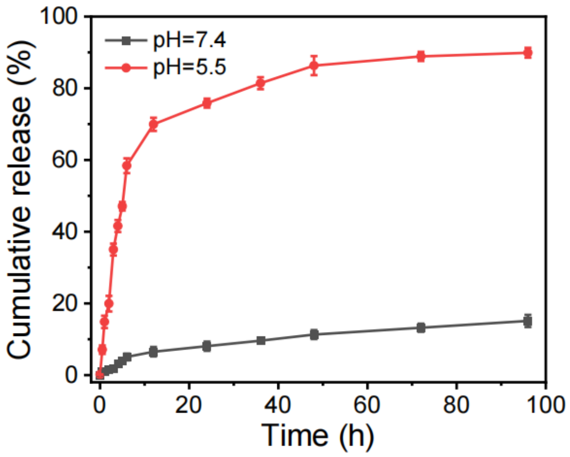

Supplementary Fig. 15 The cumulative release profiles of ICG from HIL@Z in PBS with different pH values. Data were presented as mean ± SD, n = 3 independent samples. Source data are provided as a Source Data file.

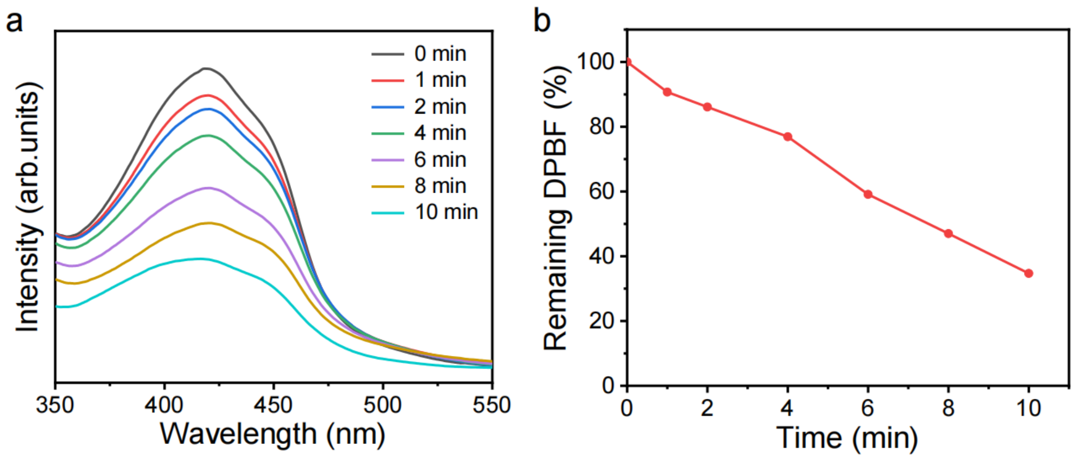

Supplementary Fig. 16 In vitro ROS generation of HIL@Z/P/H. (**a**) Time-dependent absorbance spectra and (**b**) their corresponding intensities at 410 nm of DPBF co-incubated with HIL@Z/P/H under NIR irradiation (1.5 W/cm^2). Source data are provided as a Source Data file.

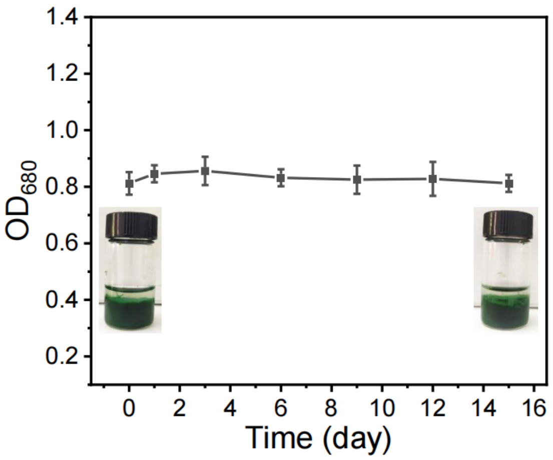

Supplementary Fig. 17 The change of absorbance intensities at 680 nm of P/H solution after storage for different days. The inset images are the corresponding P/H solution on days 0 and 15. Data were presented as mean ± SD, n = 3 independent samples. Source data are provided as a Source Data file.

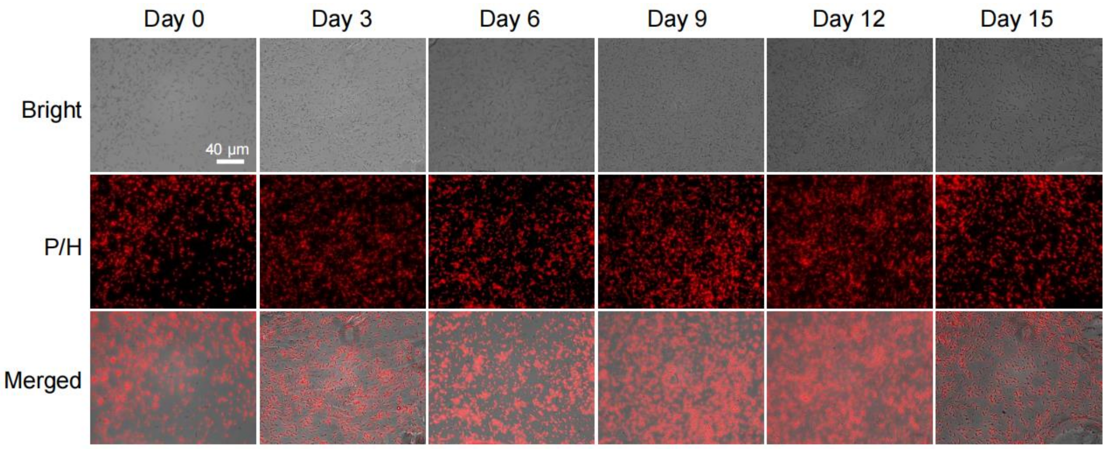

Supplementary Fig. 18 Bright-field, fluorescence and merged images of P/H after storage for different days. The results were representative of three independent experiments.

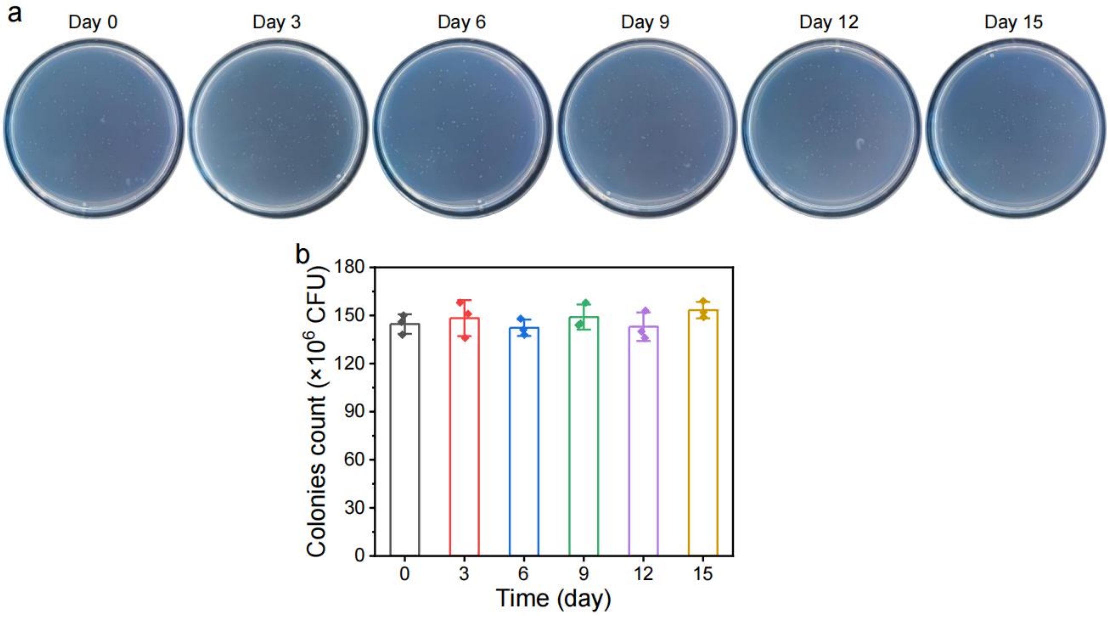

Supplementary Fig. 19 In vitro vitality characterization of PCC 7942 encapsulated in P/H. (**a**) Photographs of colonies of PCC 7942 encapsulated in the hydrogel after storage for different days. (**b**) Quantitative statistics of the number of PCC 7942 colonies through standard plate counting assay. Data in (**b**) were presented as mean ± SD, n = 3 independent samples. Source data are provided as a Source Data file.

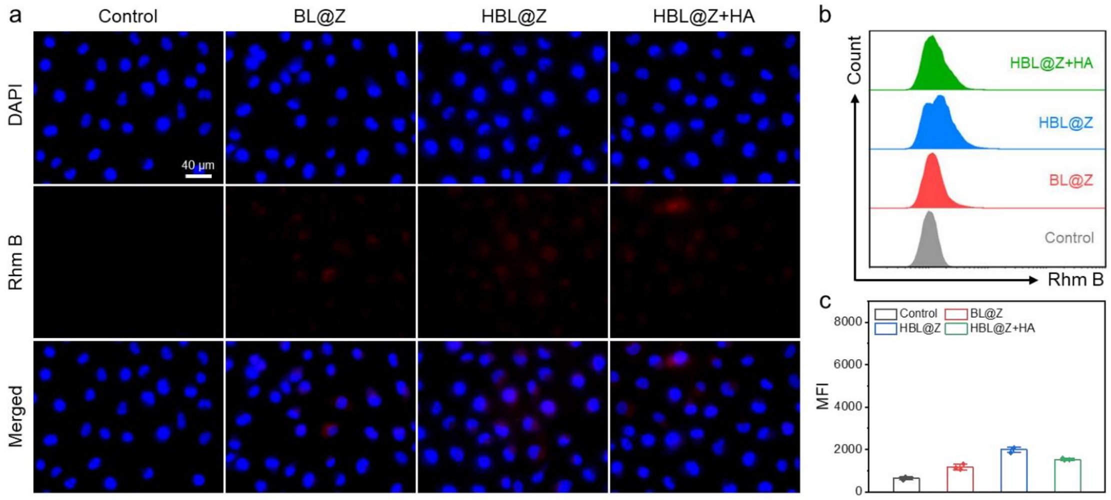

Supplementary Fig. 20 The cellular uptake of different nanoparticles by HUVECs. (**a**) Fluorescence images of HUVECs treated with different nanoparticles. (**b**) Flow cytometry analysis of Rhm B signal and (**c**) corresponding mean fluorescence intensity (MFI) in HUVECs treated with different nanoparticles. Data in (**c**) were presented as mean ± SD, n = 3 biologically independent samples. Source data are provided as a Source Data file.

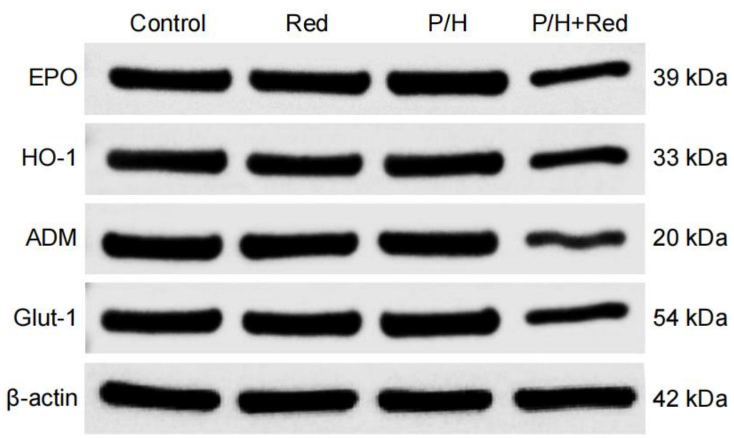

Supplementary Fig. 21 WB analysis of the expressions of EPO, HO-1, ADM and Glut-1 proteins in B16F10 cells after different treatments. Source data are provided as a Source Data file.

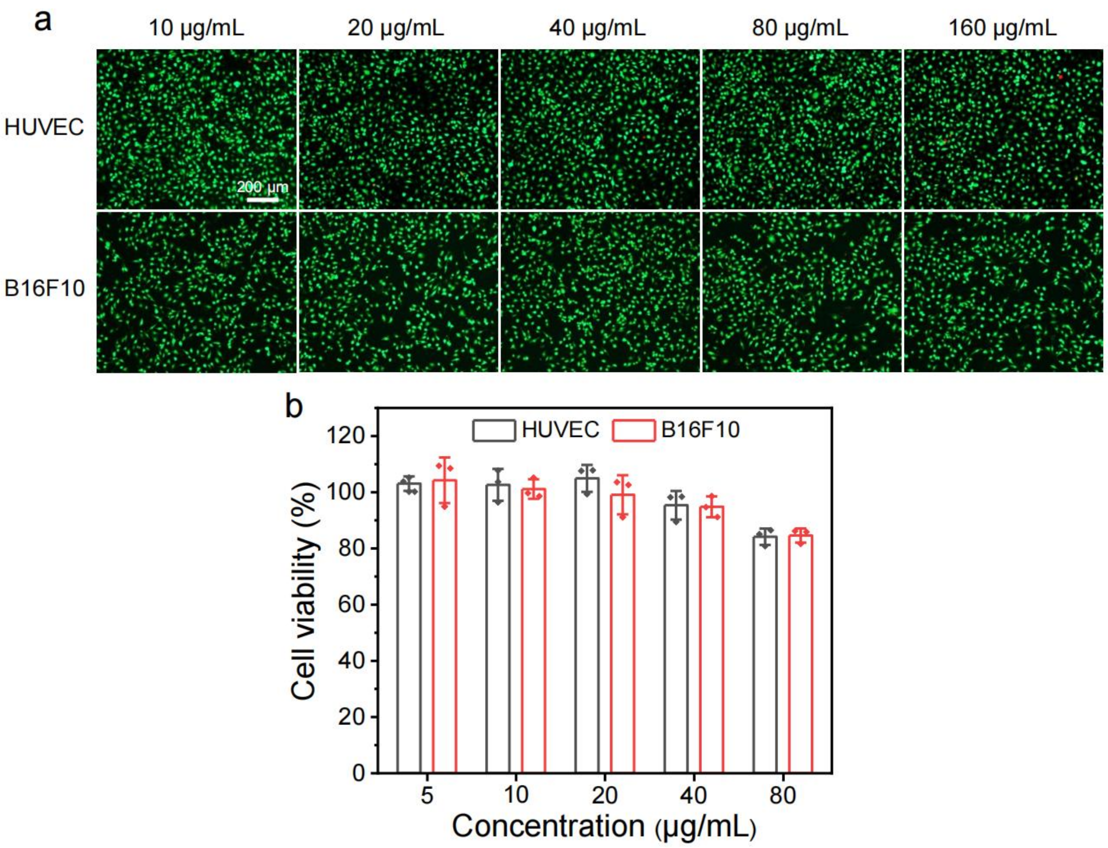

Supplementary Fig. 22 In vitro cytocompatibility of HIL@Z. (**a**) Fluorescence images of Calcein-AM/PI-stained HUVECs and B16F10 cells co-cultured with different concentrations of HIL@Z. (**b**) The cell viabilities of HUVECs and B16F10 cells co-cultured with different concentrations of HIL@Z. Data in (**b**) were presented as mean ± SD, n = 3 biologically independent samples. Source data are provided as a Source Data file.

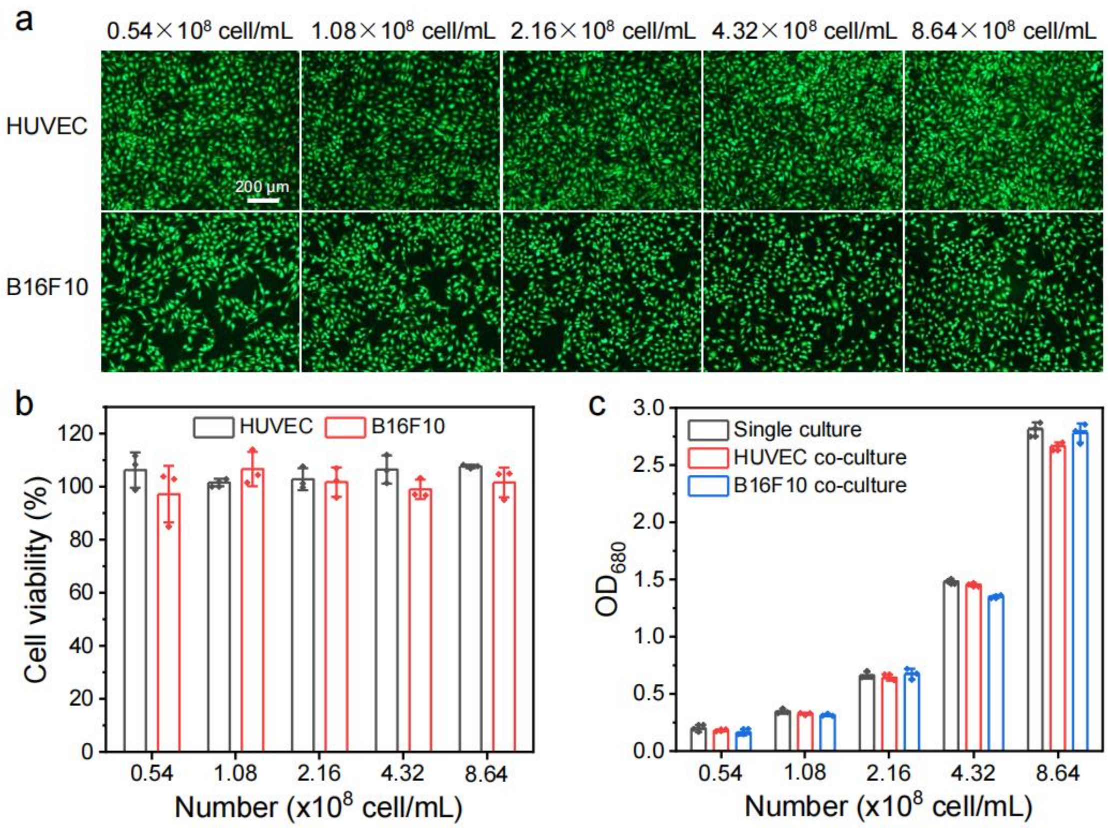

Supplementary Fig. 23 In vitro cytocompatibility of PCC 7942. (**a**) Fluorescence images of Calcein-AM/PI-stained HUVECs and B16F10 cells co-cultured with different concentrations of PCC 7942. (**b**) The cell viabilities of HUVECs and B16F10 cells co-cultured with different concentrations of PCC 7942. (**c**) Growth of PCC 7942 under different culture conditions. Data in (**b**, **c**) were presented as mean ± SD, n = 3 biologically independent samples. Source data are provided as a Source Data file.

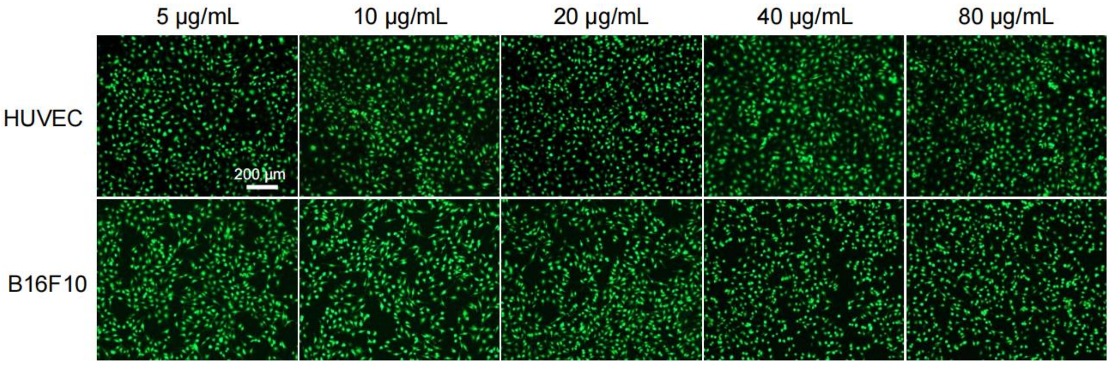

Supplementary Fig. 24 Fluorescence images of Calcein-AM/PI-stained HUVECs and B16F10 cells co-cultured with different concentrations of HIL@Z/P/H. The results were representative of three biologically independent experiments.

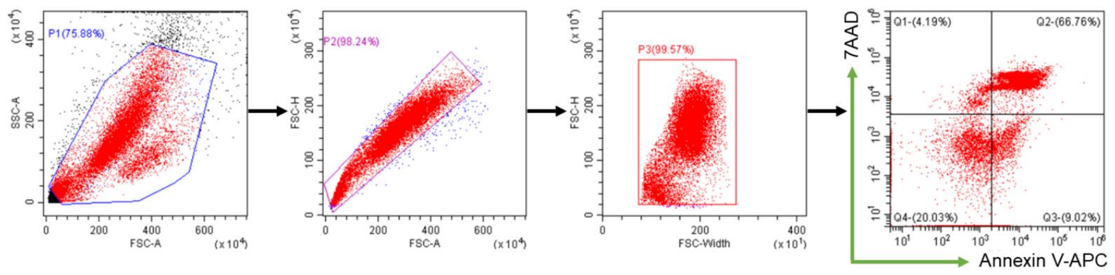

Supplementary Fig. 25 Gating strategy for flow cytometry. The experiments were repeated three times independently.

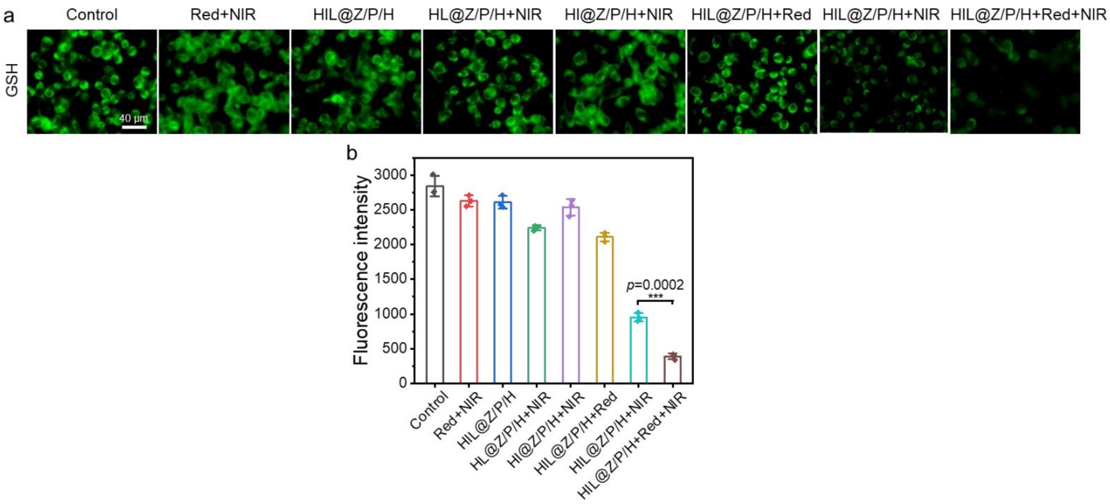

Supplementary Fig. 26 In vitro GSH depletion of HIL@Z/P/H. (**a**) Fluorescence images showing intracellular GSH detection in B16F10 cells and (**b**) their corresponding fluorescence intensities. Data in (**b**) were presented as mean ± SD, n = 3 biologically independent samples. *P* values were calculated via multiple comparisons one-way ANOVA method t-test. Source data are provided as a Source Data file.

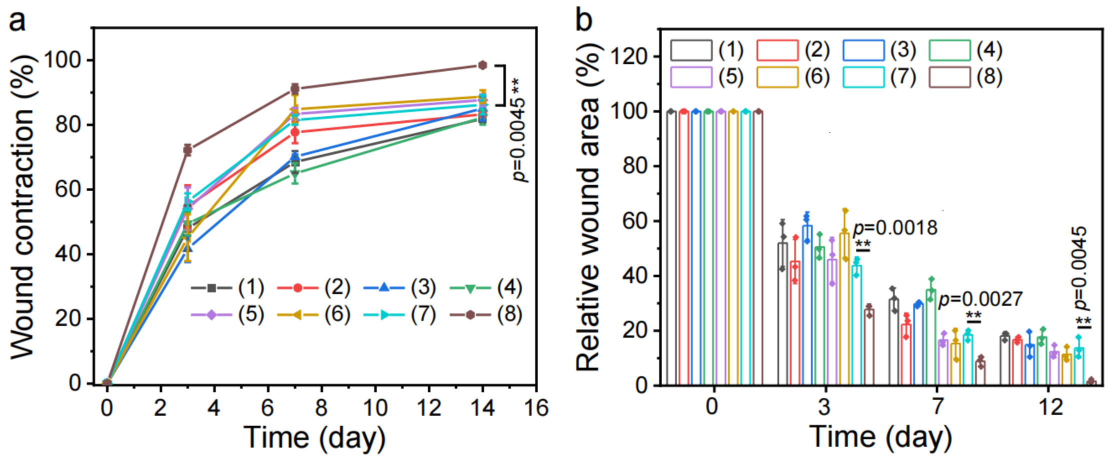

Supplementary Fig. 27 In vivo postoperative wound healing. (**a**) Quantitative analysis of dynamic wound contraction process on the wound bed in different groups. (**b**) Quantitative analysis of the wound area during recovery process in different groups. Treatments: (1) Control, (2) Red+NIR, (3) HIL@Z/P/H, (4) HL@Z/P/H+NIR, (5) HI@Z/P/H+NIR, (6) HIL@Z/P/H+Red, (7) HIL@Z/P/H+NIR, (8) HIL@Z/P/H+Red+NIR. Data were presented as mean ± SD, n = 3 biologically independent mice. *P* values were calculated via multiple comparisons one-way ANOVA method t-test. Source data are provided as a Source Data file.

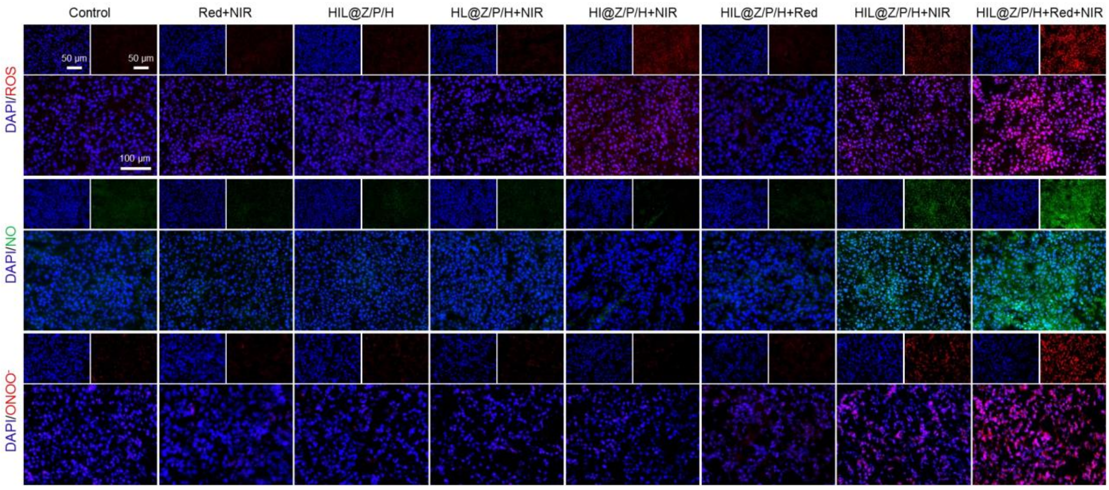

Supplementary Fig. 28 Representative ROS, NO, and ONOO$^-$ staining images of tumor tissues from mice after different treatments. The results were representative of three independent mice.

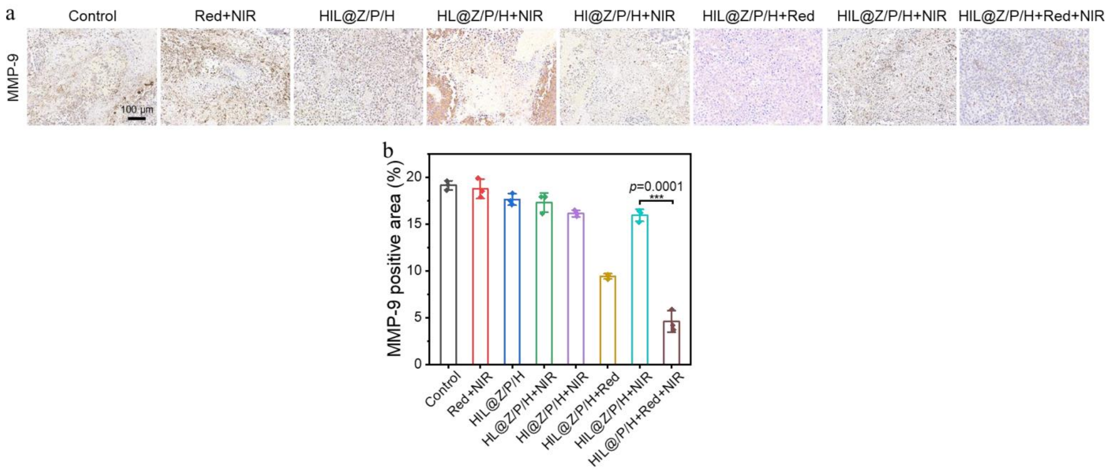

Supplementary Fig. 29 Immunohistochemistry of tumor sections. (**a**) MMP-9 staining tumor slices and (**b**) their quantitative analysis in different groups. Data in (**b**) were presented as mean ± SD, n=3 biologically independent mice. *P* values were calculated via multiple comparisons one-way ANOVA method t-test. Source data are provided as a Source Data file.

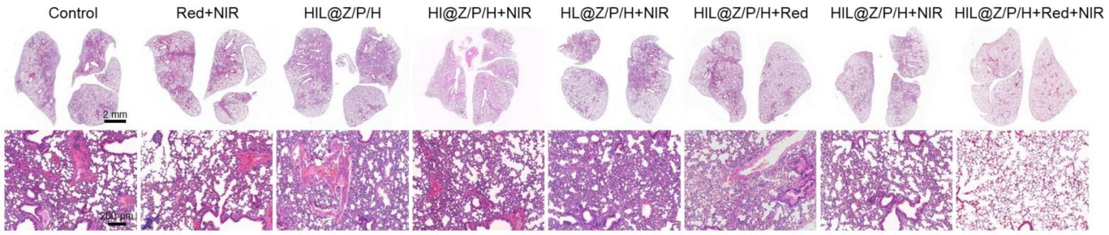

Supplementary Fig. 30 Representative images of H&E-stained lung tissues in different groups. The results were representative of three independent mice.

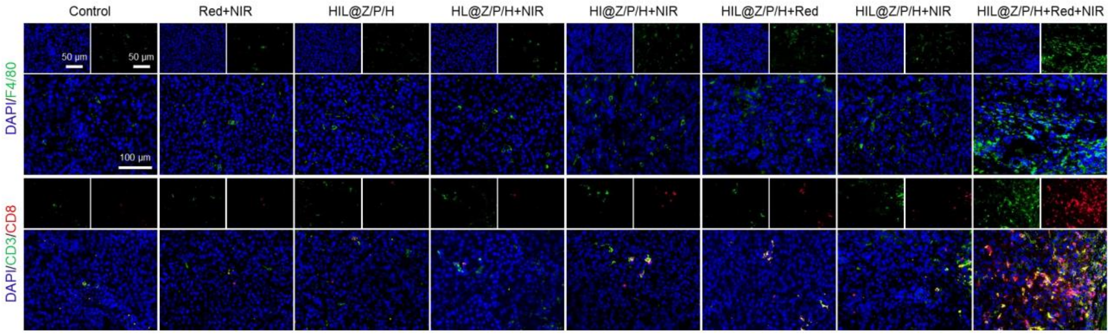

Supplementary Fig. 31 Immunofluorescence imaging of the infiltrated F4/80^{+} macrophages and CD3^{+}CD8^{+} cells in the tumor tissues from different groups. The results were representative of three independent mice.

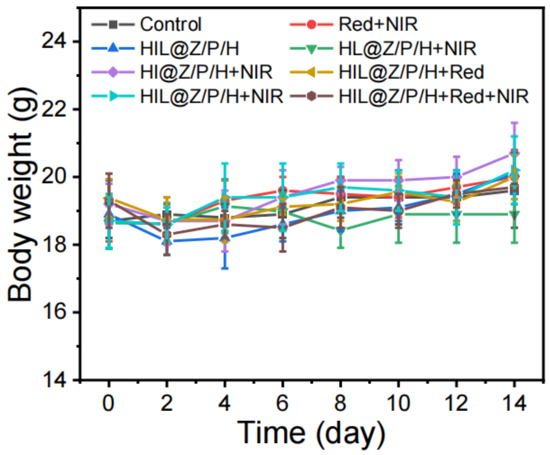

Supplementary Fig. 32 Changes of mice body weights in different groups during the 14-day treatment period. Data were presented as mean ± SD, n=5 biologically independent mice. Source data are provided as a Source Data file.

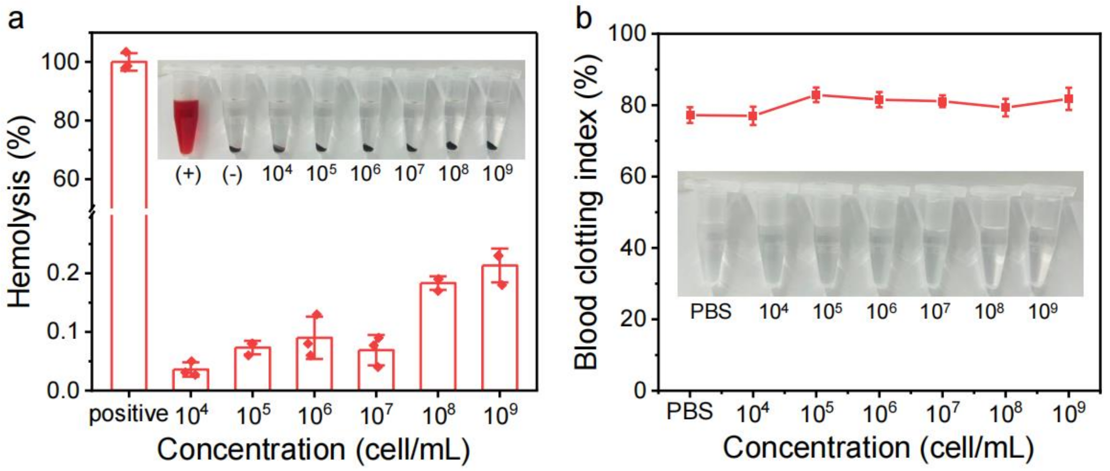

Supplementary Fig. 33 Hemolysis and coagulation of HIL@Z/P/H. (**a**) Hemolytic percent of RBCs incubated with HIL@Z/P/H with various concentrations. Triton X-100 (0.1%) and PBS were used as positive and negative controls, respectively. Inset: images for direct observation of hemolysis. (**b**) Blood clotting index changes of HIL@Z/P/H with various concentrations. DI water and PBS were used as positive and negative controls, respectively. Inset: images showing the supernatant of whole blood after incubation with HIL@Z/P/H with various concentrations. Data were presented as mean ± SD, n=3 biologically independent mice. Source data are provided as a Source Data file.

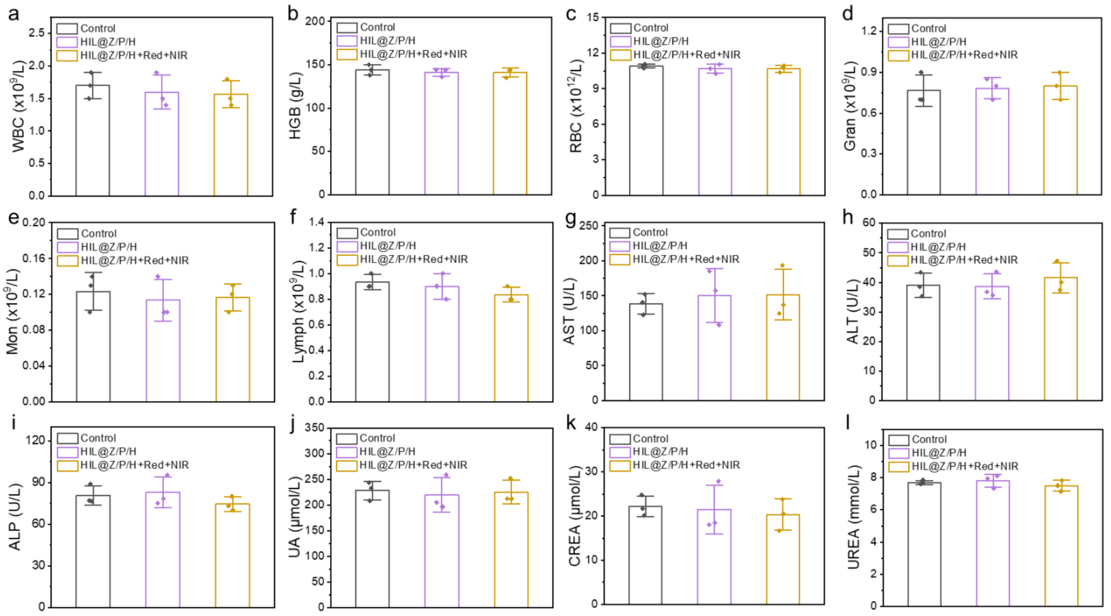

Supplementary Fig. 34 Routine blood and biochemical functions of HIL@Z/P/H. (a-f) Hematology parameters and (**g-l**) blood biochemical indexes of mice after different treatments. Mice treated with PBS solution served as control. Data were presented as mean ± SD, n=3 biologically independent mice. Source data are provided as a Source Data file.

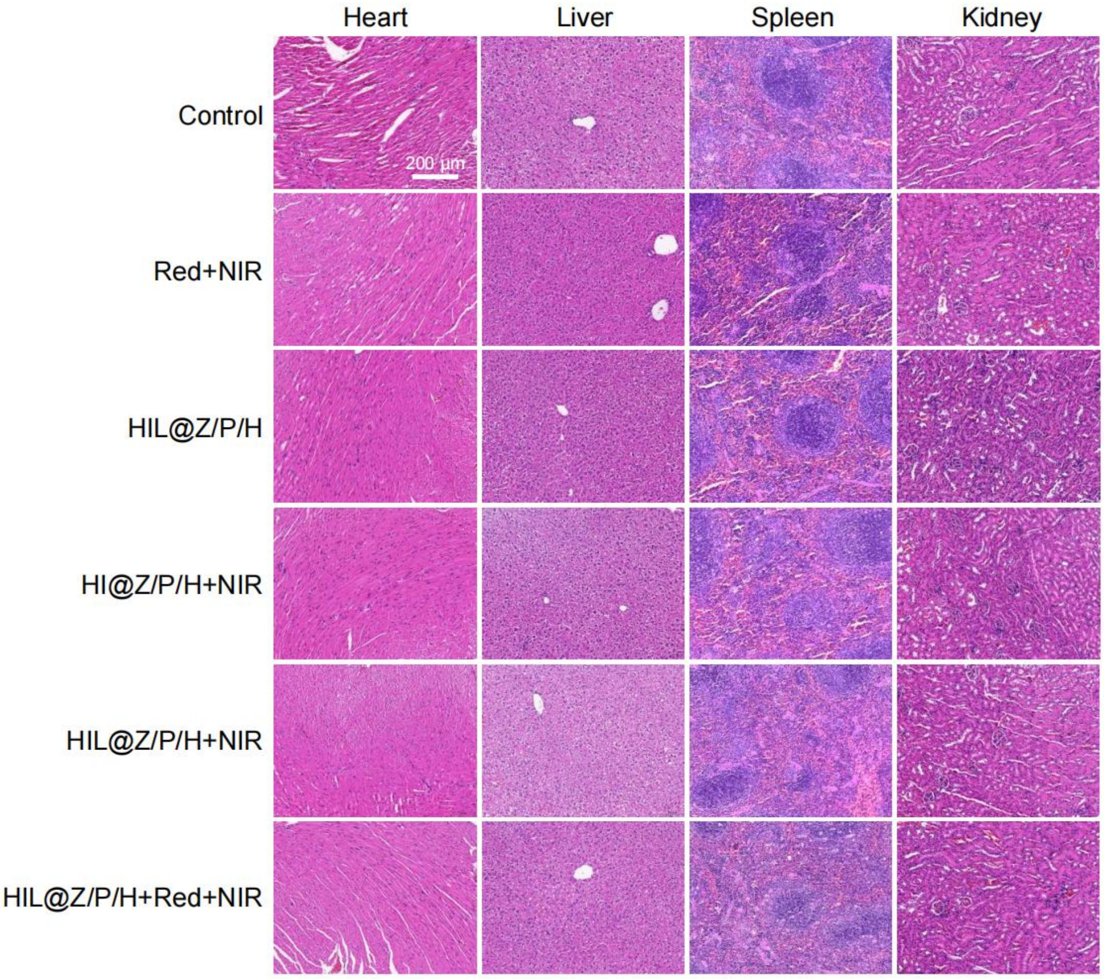

Supplementary Fig. 35 H&E staining of tissue sections from normal organs harvested from B16F10 tumor-bearing mice on the 14th day from different groups. The results were representative of three independent mice.

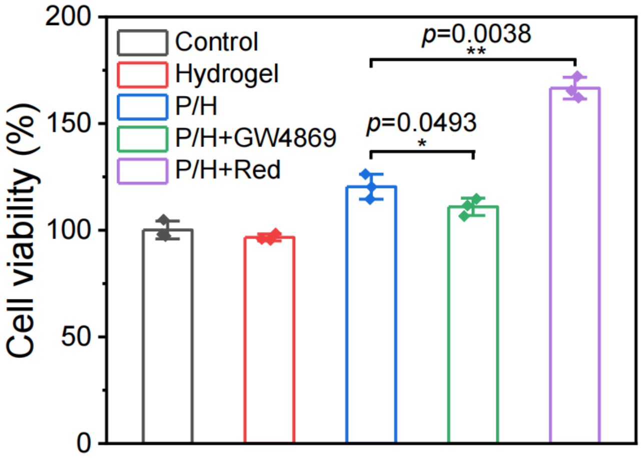

Supplementary Fig. 36 Alamar blue assay of HUVECs proliferation in different groups. Data were presented as mean ± SD, n = 3 biologically independent samples. *P* values were calculated via multiple comparisons one-way ANOVA method t-test. Source data are provided as a Source Data file.

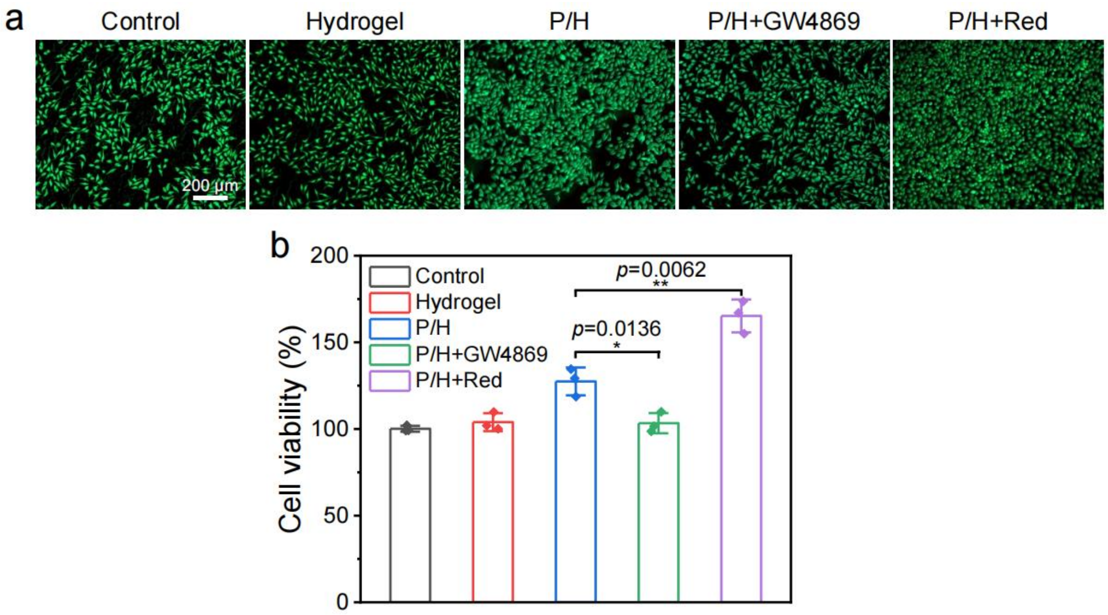

Supplementary Fig. 37 In vitro proliferation of L929 cells in different groups. (**a**) Live/dead-staining images of L929 cells cultured in different groups. (**b**) Living cell ratio of L929 cells cultured in different groups. Data in (**b**) were presented as mean ± SD, n = 3 biologically independent samples. *P* values were calculated via multiple comparisons one-way ANOVA method t-test. Source data are provided as a Source Data file.

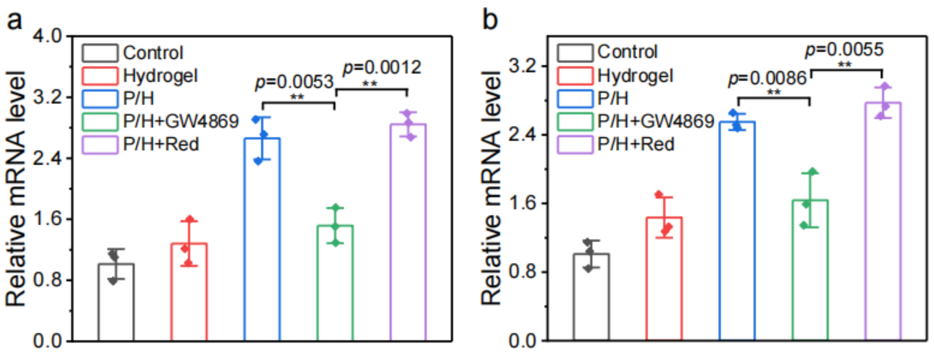

Supplementary Fig. 38 In vitro expression of *IL-6* mRNA in different groups. RT-qPCR analysis of the expression of *IL-6* mRNA in (**a**) HUVECs and (**b**) L929 cells after different treatments. Data were presented as mean ± SD, n = 3 biologically independent samples. *P* values were calculated via multiple comparisons one-way ANOVA method t-test. Source data are provided as a Source Data file.

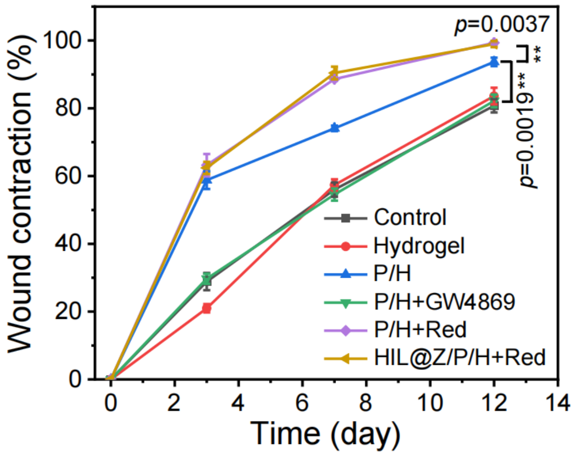

Supplementary Fig. 39 Quantitative analysis of dynamic wound contraction process on the wound bed in different groups. Data were presented as mean ± SD, n = 3 biologically independent mice. *P* values were calculated via multiple comparisons one-way ANOVA method t-test. Source data are provided as a Source Data file.

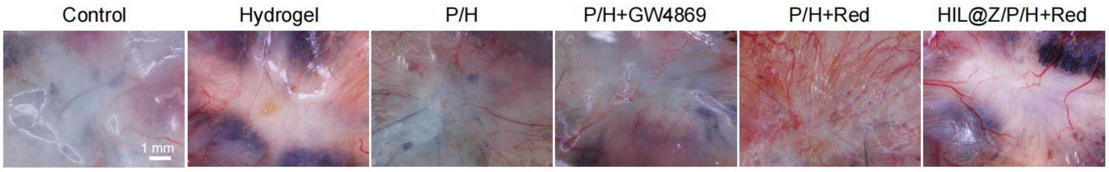

Supplementary Fig. 40 Representative photographs of angiogenesis on the wound bed in different groups. The results were representative of three independent mice.

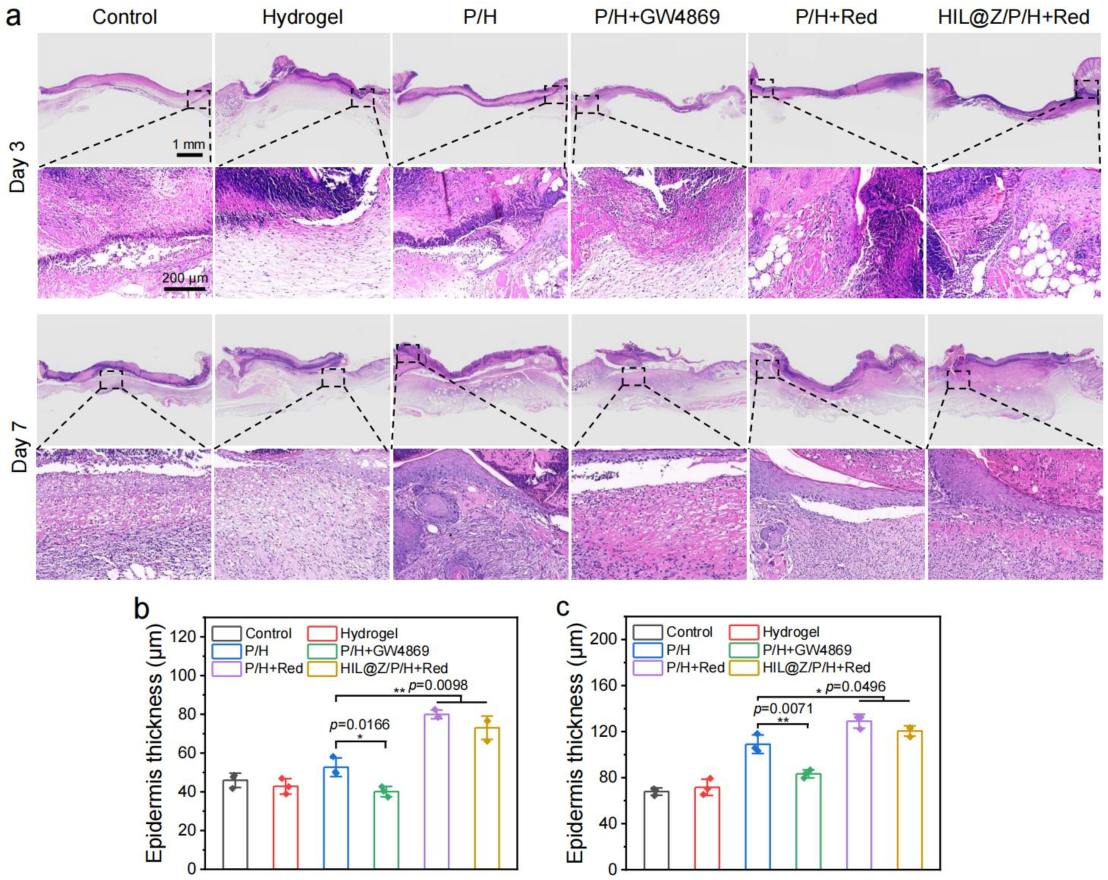

Supplementary Fig. 41 H&E staining of wound sections in different groups. (**a**) H&E staining of the wounds in different groups on days 3 and 7. Quantification of the epithelial thickness in different groups on days 3 (**b**) and 7 (**c**). Data in (**b**, **c**) were presented as mean ± SD, n=3 biologically independent mice. *P* values were calculated via multiple comparisons one-way ANOVA method t-test. Source data are provided as a Source Data file.

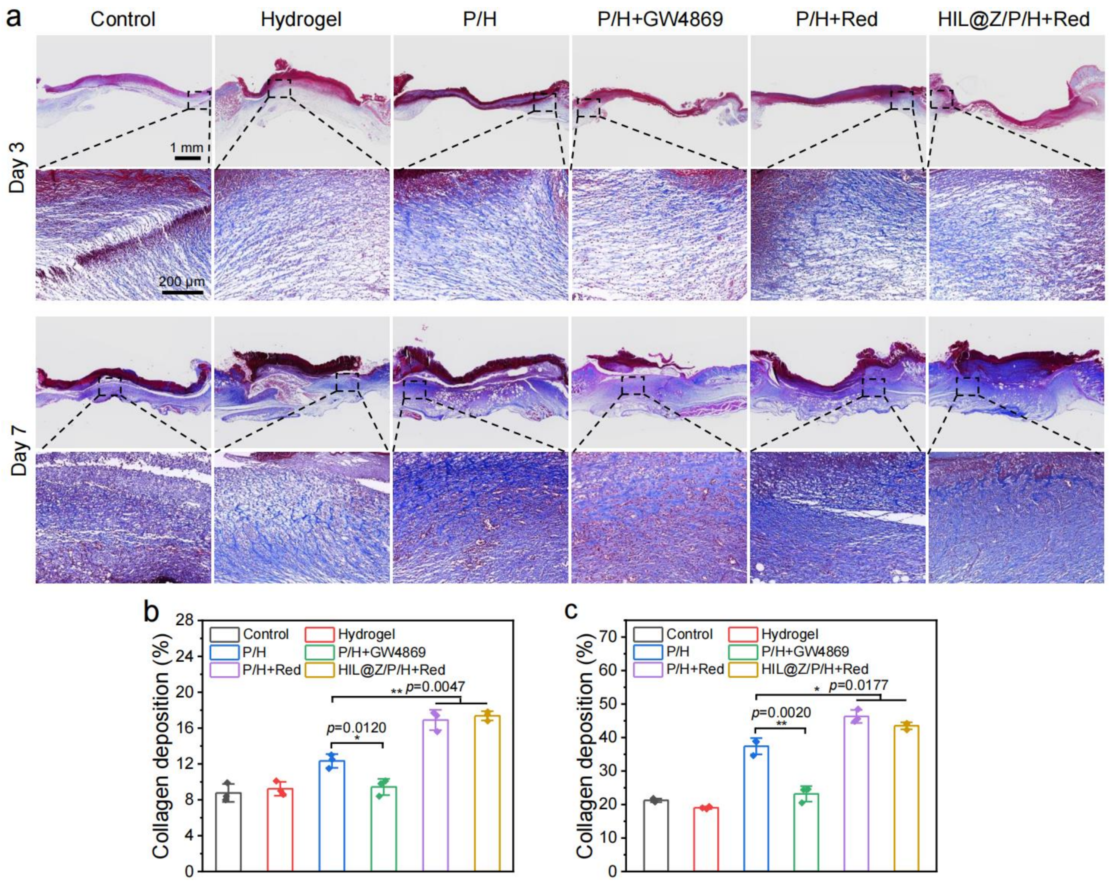

Supplementary Fig. 42 Masson staining of wound sections in different groups. (**a**) Masson staining of the wounds in different groups on days 3 and 7. Quantification of the collagen deposition in different groups on days 3 (**b**) and 7 (**c**). Data in (**b**, **c**) were presented as mean ± SD, n=3 biologically independent mice. *P* values were calculated via multiple comparisons one-way ANOVA method t-test. Source data are provided as a Source Data file.

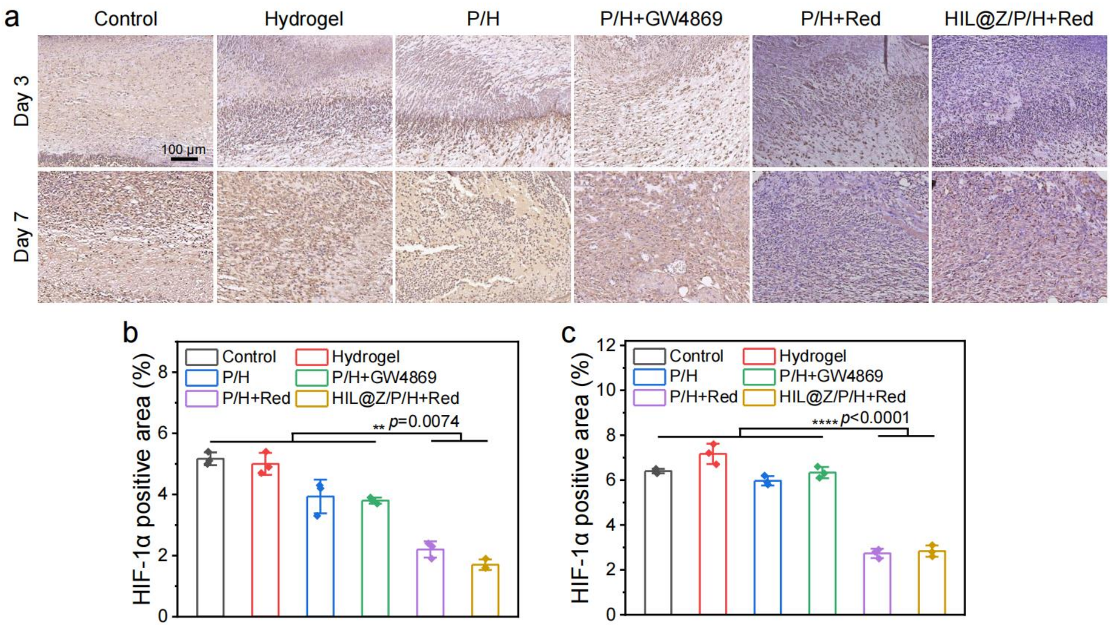

Supplementary Fig. 43 HIF-1α staining of wound tissues in different groups. (**a**) HIF-1α staining of wound tissues in different groups on days 3 and 7. Quantitative analysis of HIF-1α-positive area in different groups on days 3 (**b**) and 7 (**c**). Data in (**b**, **c**) were presented as mean ± SD, n=3 biologically independent mice. *P* values were calculated via multiple comparisons one-way ANOVA method t-test. Source data are provided as a Source Data file.

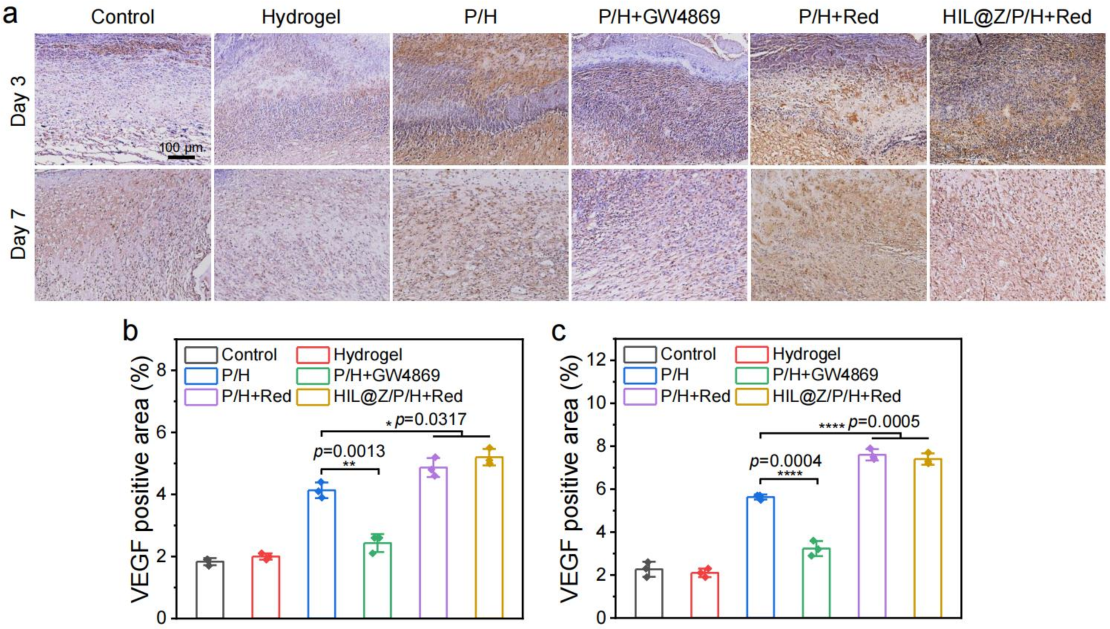

Supplementary Fig. 44 VEGF staining of wound tissues in different groups. (**a**) VEGF staining of wound tissues in different groups on days 3 and 7. Quantitative analysis of VEGF-positive area in different groups on days 3 (**b**) and 7 (**c**). Data in (**b**, **c**) were presented as mean ± SD, n=3 biologically independent mice. *P* values were calculated via multiple comparisons one-way ANOVA method t-test. Source data are provided as a Source Data file.

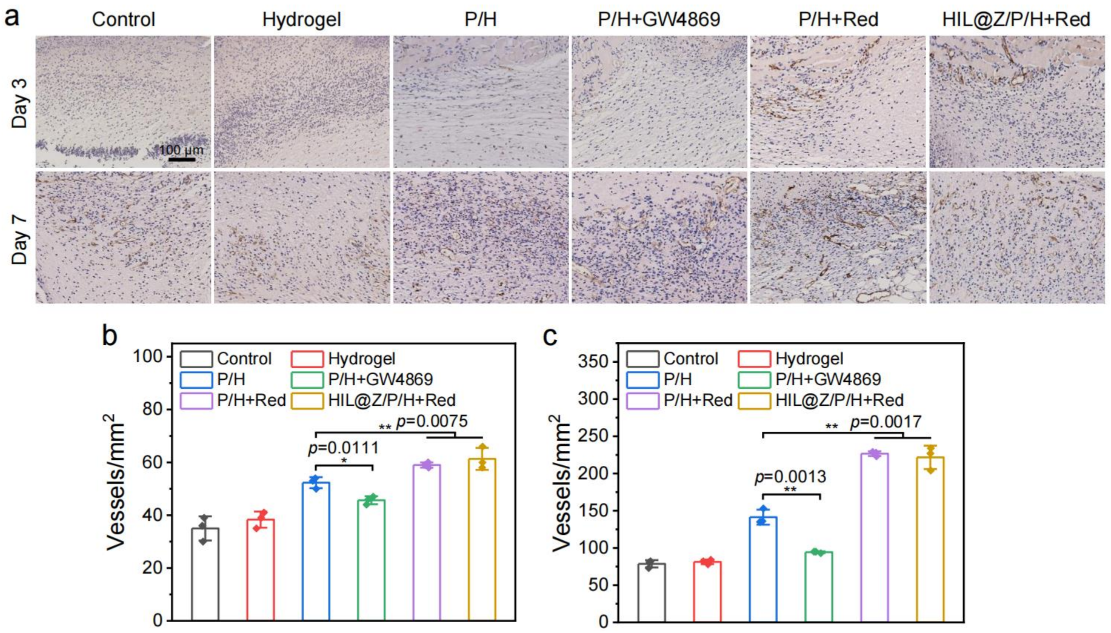

Supplementary Fig. 45 CD31 staining of wound tissues in different groups. (**a**) CD31 staining of wound tissues in different groups on days 3 and 7. Quantitative analysis of blood vessels in regenerated dermis from different groups on days 3 (**b**) and 7 (**c**). Data in (**b**, **c**) were presented as mean ± SD, n=3 biologically independent mice. *P* values were calculated via multiple comparisons one-way ANOVA method t-test. Source data are provided as a Source Data file.

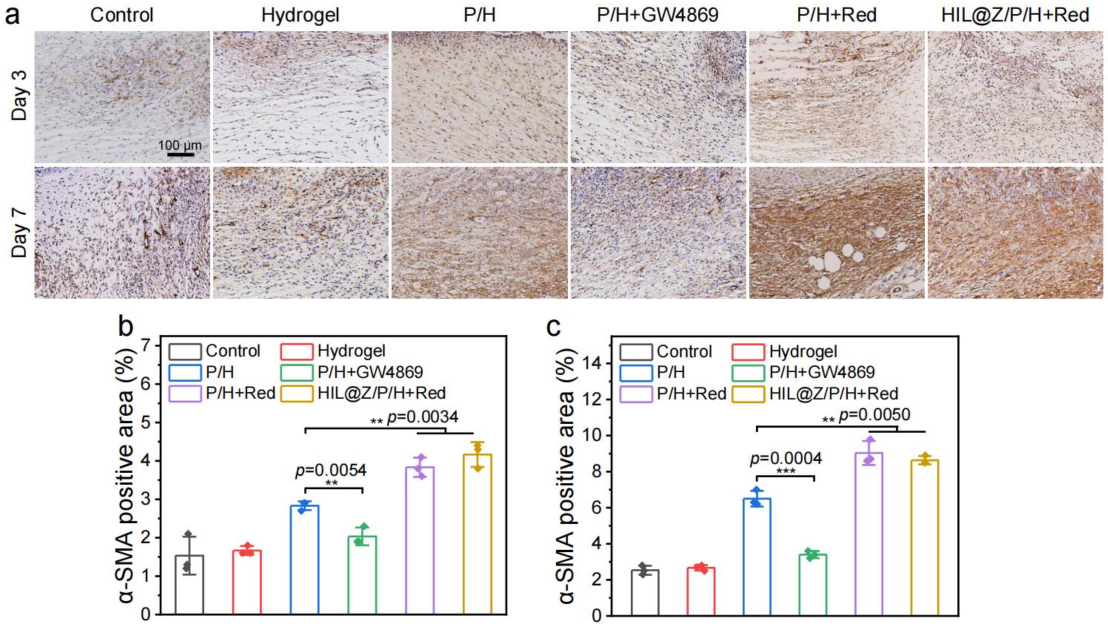

Supplementary Fig. 46 α-SMA staining of wound tissues in different groups. (**a**) α-SMA staining of wound tissues in different groups on days 3 and 7. Quantitative analysis of α-SMA-positive area in different groups on days 3 (**b**) and 7 (**c**). Data in (**b**, **c**) were presented as mean ± SD, n=3 biologically independent mice. *P* values were calculated via multiple comparisons one-way ANOVA method t-test. Source data are provided as a Source Data file.
