# Supplementary material for: In-situ-sprayed therapeutic hydrogel for oxygen-actuated Janus regulation of postsurgical tumor recurrence/metastasis and wound healing
Source: Nat Commun. 2024 Jan 27;15:814. doi: 10.1038/s41467-024-45072-x (PMC10821930; doi:10.1038/s41467-024-45072-x)
Supplement: Supplementary file 2 — Reporting Summary [file 41467_2024_45072_MOESM2_ESM.pdf]

nature portfolio

Corresponding author(s): Jianwen Hou, Shaobing Zhou

Last updated by author(s): Dec 30, 2023

Reporting Summary

Nature Portfolio wishes to improve the reproducibility of the work that we publish. This form provides structure for consistency and transparency in reporting. For further information on Nature Portfolio policies, see our Editorial Policies and the Editorial Policy Checklist.

Statistics

For all statistical analyses, confirm that the following items are present in the figure legend, table legend, main text, or Methods section.

n/a	Confirmed	
☐	☒	The exact sample size (*n*) for each experimental group/condition, given as a discrete number and unit of measurement
☐	☒	A statement on whether measurements were taken from distinct samples or whether the same sample was measured repeatedly
☐	☒	The statistical test(s) used AND whether they are one- or two-sided<br>*Only common tests should be described solely by name; describe more complex techniques in the Methods section.*
☒	☐	A description of all covariates tested
☒	☐	A description of any assumptions or corrections, such as tests of normality and adjustment for multiple comparisons
☐	☒	A full description of the statistical parameters including central tendency (e.g. means) or other basic estimates (e.g. regression coefficient) AND variation (e.g. standard deviation) or associated estimates of uncertainty (e.g. confidence intervals)
☐	☒	For null hypothesis testing, the test statistic (e.g. *F*, *t*, *r*) with confidence intervals, effect sizes, degrees of freedom and *P* value noted<br>*Give P values as exact values whenever suitable.*
☒	☐	For Bayesian analysis, information on the choice of priors and Markov chain Monte Carlo settings
☒	☐	For hierarchical and complex designs, identification of the appropriate level for tests and full reporting of outcomes
☒	☐	Estimates of effect sizes (e.g. Cohen's *d*, Pearson's *r*), indicating how they were calculated

Our web collection on statistics for biologists contains articles on many of the points above.

Software and code

Policy information about availability of computer code

Data collection	The sample morphologies were characterized by transmission electron microscopy (TEM) (JEM-2100F, Japan) and scanning electron microscopy (SEM) (JSM 7800F, Japan).<br>The hydrodynamic size and surface zeta potential were tested by dynamic light scattering (DLS) (ZETA-SIZER, MALVERN NanoZS90, Malvern, Ltd., UK).<br>The ultraviolet-visible (UV-vis) absorption spectra were measured by UV-2550 spectrometer (Shimadzu, Japan).<br>The chemical composition was characterized with fourier transform infrared spectroscopy (FT-IR, Fluke, Ti480).<br>The crystalline structure of nanoparticles was analyzed using Empyrean X-ray diffractometer (PANalytical B.V., NED).<br>Fluorescence spectrum was recorded by an RF-7000 fluorescence spectrophotometer (Shimadzu, Kyoto, Japan).<br>The concentrations of dissolved O2 measurements were acquired by a dissolved O2 meter (Rex, JPBJ-608).<br>TGA was performed on a thermal analyzer (Mettler Toledo TGA/DSC 3+, Switzerland).
Data analysis	The data anlaysis were performed using OriginPro 2021 (version 9.8.0.200). Flow cytometric analysis were performed using FlowJo (version 7.6.1). Histological analysis was performed using Image J (version 2.1.0).

For manuscripts utilizing custom algorithms or software that are central to the research but not yet described in published literature, software must be made available to editors and reviewers. We strongly encourage code deposition in a community repository (e.g. GitHub). See the Nature Portfolio guidelines for submitting code & software for further information.

Data

Policy information about availability of data

All manuscripts must include a data availability statement. This statement should provide the following information, where applicable:

- Accession codes, unique identifiers, or web links for publicly available datasets
- A description of any restrictions on data availability
- For clinical datasets or third party data, please ensure that the statement adheres to our policy

All data are available in the main text, Supplementary Information, or Source Data file. Source data are provided with this paper. If any raw data files are needed in another format, they are available from the corresponding author upon request.

Research involving human participants, their data, or biological material

Policy information about studies with human participants or human data. See also policy information about sex, gender (identity/presentation), and sexual orientation and race, ethnicity and racism.

Reporting on sex and gender	n/a
Reporting on race, ethnicity, or other socially relevant groupings	n/a
Population characteristics	n/a
Recruitment	n/a
Ethics oversight	n/a

Note that full information on the approval of the study protocol must also be provided in the manuscript.

Field-specific reporting

Please select the one below that is the best fit for your research. If you are not sure, read the appropriate sections before making your selection.

[x] Life sciences [ ] Behavioural & social sciences [ ] Ecological, evolutionary & environmental sciences

For a reference copy of the document with all sections, see nature.com/documents/nr-reporting-summary-flat.pdf

Life sciences study design

All studies must disclose on these points even when the disclosure is negative.

Sample size	All data were obtained from a minimum of three independent experiments and were expressed as mean ± standard deviation (SD). The statistical significance was evaluated by the student's t-test.
Data exclusions	No data was excluded from this study.
Replication	All experiments were performed with independent replicates. At least three independent samples were performed for each experiment.
Randomization	The samples were divided into different groups randomly in all experiments.
Blinding	Formal blinding was used for H&E and immunohistochemical staining of tumor and wound tissues.

Reporting for specific materials, systems and methods

We require information from authors about some types of materials, experimental systems and methods used in many studies. Here, indicate whether each material, system or method listed is relevant to your study. If you are not sure if a list item applies to your research, read the appropriate section before selecting a response.

Materials & experimental systems

n/a	Involved in the study
	☒ Antibodies
	☒ Eukaryotic cell lines
☒	Palaeontology and archaeology
	☒ Animals and other organisms
☒	Clinical data
☒	Dual use research of concern
☒	Plants

Methods

n/a	Involved in the study
☒	ChIP-seq
	☒ Flow cytometry
☒	MRI-based neuroimaging

Antibodies

Antibodies used	Anti-HIF-1 alpha (mgc3) mouse monoclonal antibodies (Catalog # ab16066), Anti-VEGF Receptor 2 (SP123) rabbit monoclonal antibody (Catalog # ab115805), Anti-CD31 (EPR17259) rabbit monoclonal antibody (Catalog # ab182981), Anti-alpha smooth muscle Actin (1A4) mouse monoclonal antibodies (Catalog # ab7817), Anti-EPOP/C17orf96 (EPR24906-32) rabbit monoclonal antibody (Catalog # ab271007), Anti-Heme Oxygenase 1 (EPR18161-128) rabbit monoclonal antibody (Catalog # ab189491), Anti-CD8 alpha (EPR21769) rabbit monoclonal antibody (Catalog # ab 217344), and Anti-Glucose Transporter GLUT1 (EPR3915) rabbit monoclonal antibody (Catalog # ab115730) were purchased from Abcam (Cambridge,UK). Adrenomedullin Polyclonal antibody (Catalog # 10778-1-AP) and F4/80 Polyclonal antibody (Catalog # 28463-1-AP) were purchased from Proteintech Group, Inc. Anti -CD3 Rabbit pAb (Catalog # GB11014-100) and Anti -MMP9 Rabbit pAb (Catalog # GB11132-100) were purchased from Servicebio. Anti-beta Actin Rabbit pAb (Catalog # 380624) were purchased from ZENBIO. Goat Anti-Rabbit IgG HRP (Catalog # BL003A) was purchased from Biosharp.
Validation	Validation details of the primary antibodies are available on the manufacturers' websites:<br>https://www.abcam.cn/products/primary-antibodies/hif-1-alpha-antibody-mgc3-ab16066.html<br>https://www.abcam.cn/products/primary-antibodies/vegf-receptor-2-antibody-sp123-ab115805.html<br>https://www.abcam.cn/products/primary-antibodies/cd31-antibody-epr17259-ab182981.html<br>https://www.abcam.cn/products/primary-antibodies/alpha-smooth-muscle-actin-antibody-1a4-ab7817.html<br>https://www.abcam.cn/products/primary-antibodies/epopc17orf96-antibody-epr24906-32-ab271007.html<br>https://www.abcam.cn/products/primary-antibodies/heme-oxygenase-1-antibody-epr18161-128-ab189491.html<br>https://www.abcam.cn/products/primary-antibodies/glucose-transporter-glut1-antibody-epr3915-ab115730.html<br>https://www.abcam.cn/products/primary-antibodies/cd8-alpha-antibody-epr21769-ab217344.html<br>https://www.ptgcn.com/products/ADM-Antibody-10778-1-AP.htm<br>https://www.ptgcn.com/products/F4-80-Antibody-28463-1-AP.htm<br>https://www.servicebio.cn/goodsdetail?id=1319<br>https://www.servicebio.cn/goodsdetail?id=1401<br>http://www.zen-bio.cn/prod_view.aspx?IsActiveTarget=True&TypeId=179&Id=535567&FId=t3:179:3<br>http://www.cmorn.com/ProductDetail/4096598.html

Eukaryotic cell lines

Policy information about cell lines and Sex and Gender in Research

Cell line source(s)	Human umbilical vein endothelial cells (HUVECs), mouse melanoma cells (B16F10) and mouse fibroblasts (L929) were purchased from the Cell Bank of Chinese Academy of Sciences (Shanghai, China).
Authentication	All cell lines were authenticated by the supplier using Short Tandem Repeat test.
Mycoplasma contamination	It is negative for mycoplasma.
Commonly misidentified lines (See ICLAC register)	No commonly misidentified cell lines were used.

Animals and other research organisms

Policy information about studies involving animals; ARRIVE guidelines recommended for reporting animal research, and Sex and Gender in Research

Laboratory animals	Specific pathogen-free (SPF) C57BL/6 mice (female, 6-8 week) were purchased from Chengdu Dossy Experimental Animals Co., Ltd.
Wild animals	The study did not involve wild animals.
Reporting on sex	Female mice were used in this study.
Field-collected samples	No field collected samples were used in the study.

Ethics oversight | All animal operations and experimental procedures were approved by the Institutional Animal Care and Use Committee of Southwest Jiaotong University.

Note that full information on the approval of the study protocol must also be provided in the manuscript.

Plants

Seed stocks | n/a

Novel plant genotypes | n/a

Authentication | n/a

Flow Cytometry

Plots

Confirm that:

- [x] The axis labels state the marker and fluorochrome used (e.g. CD4-FITC).
- [x] The axis scales are clearly visible. Include numbers along axes only for bottom left plot of group (a 'group' is an analysis of identical markers).
- [x] All plots are contour plots with outliers or pseudocolor plots.
- [x] A numerical value for number of cells or percentage (with statistics) is provided.

Methodology

Sample preparation | All assays were performed according to the manufacturer's instructions. Dihydrorhodamine 123 (DHR123) (Catalog # 109244-58-8) were obtained from Aladdin Industrial Corporation. 2,7-dichlorodihydrofluorescein diacetate (DCFH-DA) (Catalog # S0033S) and NO fluorescent probe (DAF-FM DA) (Catalog # S0019S) were obtained from Beyotime Biotechnology. The Annexin V-PE/7-AAD apoptosis kit (Catalog # 70-AT101-100) were purchased from MultiSciences.
Validation details reagent kits are available on the manufacturers' websites:
https://www.aladdin-e.com/zh_cn/d115501.html
https://www.beyotime.com/product/S0033S.htm
https://www.beyotime.com/product/S0019S.htm
https://www.liankebio.com/product/annexin-v-pe-7-aad-apoptosis-kit-12744.html

Instrument | Coulter FC-500 flow cytometer

Software | FlowJo 7.6.1.

Cell population abundance | The instrument counts 10000 to 50000 cells autonomously.

Gating strategy | All samples were gated equally.

- [x] Tick this box to confirm that a figure exemplifying the gating strategy is provided in the Supplementary Information.
