# Supplementary material for: In-situ-sprayed therapeutic hydrogel for oxygen-actuated Janus regulation of postsurgical tumor recurrence/metastasis and wound healing
Source: Nat Commun. 2024 Jan 27;15:814. doi: 10.1038/s41467-024-45072-x (PMC10821930; doi:10.1038/s41467-024-45072-x)
Supplement: Supplementary file 3 — Description of Additional Supplementary Files [file 41467_2024_45072_MOESM3_ESM.pdf]

Description of Additional Supplementary Files

File Name: Supplementary Movie 1

In situ formation process of the sprayed HIL@Z/P/H hydrogel at the postoperative wound site.
